# The Hierarchical Trimetallic FeCoNi LTH/MnMoO_4_/GO Nanohybrid as an Active Catalyst with Enhanced Bifunctional Performance for Efficient Overall Water Splitting

**DOI:** 10.1021/acsami.5c20919

**Published:** 2026-03-07

**Authors:** Fahimeh Sadat Vajedi, Yutao Xing, Nakédia M. F. Carvalho

**Affiliations:** a Universidade do Estado do Rio de Janeiro, Instituto de Química, Rua São Francisco Xavier, 524, Maracanã, Rio de Janeiro, RJ 20550-013, Brazil; b Universidade Federal Fluminense, Instituto de Física, Ave. Gal. Milton Tavares de Souza, Campus Praia Vermelha, São Domingos, Nitéroi 24210-346, Brazil

**Keywords:** FeCoNi layered triple hydroxide, MnMoO_4_, graphene oxide, bifunctional electrocatalyst, overall water splitting

## Abstract

The development of efficient and stable bifunctional electrocatalysts based on Earth-abundant elements for sustainable hydrogen production via water splitting is of significant importance. In this study, we present the design and synthesis of a novel and highly efficient electrode based on trimetallic FeCoNi layered triple hydroxide (LTH) nanoparticles decorated over MnMoO_4_ nanorods and graphene oxide (GO) electrodeposited onto fluorine-doped tin oxide (FTO) substrates. The heterostructured interface among the FeCoNi LTH nanoparticles, MnMoO_4_ nanorods, and GO also offers more available catalytic sites, enhances electronic interactions, and accelerates the kinetics of water dissociation. As a result, the FeCoNi LTH/MnMoO_4_/GO/FTO electrocatalyst shows improved bifunctional electrocatalytic activity toward both the oxygen evolution reaction (OER) and hydrogen evolution reaction (HER), delivering a low overpotential of 238 mV for the OER and 92 mV for the HER at a current density of ±10 mA cm^–2^, along with small Tafel slopes of 53 and 46 mV dec^–1^, respectively. Furthermore, the electrocatalyst preserves outstanding stability over 31 h of continuous electrolysis with an overall water-splitting voltage of 1.57 V at 10 mA cm^–2^, surpassing the performance of commercial RuO_2_-based systems. This work presents a novel method for constructing high-performance and bifunctional electrocatalysts based on Earth-abundant elements for efficient water electrolysis, offering a promising pathway toward cost-effective large-scale hydrogen production.

## Graphical abstract

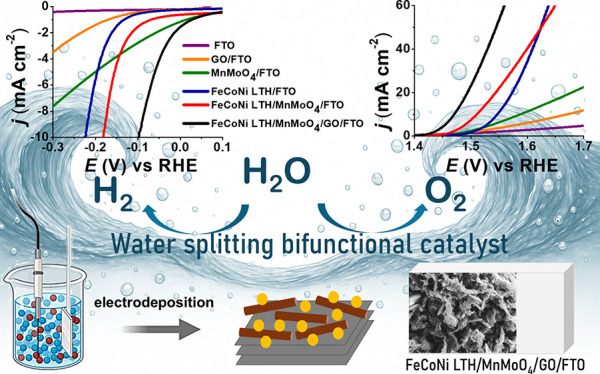

## 1 Introduction

The urgency to confront the energy scarcity and environmental challenges arising from fossil fuel consumption has stimulated significant scientific progress into sustainable energy conversion and storage strategies.^1^ Electrochemical water splitting has emerged as a particularly attractive route for green hydrogen production^2^ due to its cost-effectiveness, operational convenience, and nonpolluting nature.^3^ Nevertheless, the slow kinetics of the hydrogen evolution reaction (HER) and, in particular, of the oxygen evolution reaction (OER) requires the development of highly efficient and stable electrocatalysts.

Benchmark electrocatalysts based on noble metals, including Pt for HER and RuO_2_/IrO_2_ for OER, demonstrate remarkable catalytic performance and long-term stability; however, their elevated cost and scarcity significantly hinder widespread use in large-scale applications.^4,5^ Consequently, recent advances in catalyst development have increasingly focused on complex multicomponent materials, particularly nanostructures incorporating transition metals such as manganese, iron, nickel, and cobalt, inspired by redox-active enzymes.^6,7^ Transition metal dichalcogenides including MoS_2_ and WS_2_, as well as phosphides,^8^ selenides, and borides, have exhibited remarkable efficiency in catalyzing HER under acidic conditions. In contrast, transition metal oxides, hydroxides, and oxyhydroxides have demonstrated favorable OER performance in alkaline electrolytes.^9^

Bifunctional catalysts that can catalyze both HER and OER are highly appealing for water splitting devices, as they simplify the system and reduce the overall costs by enabling the use of a single electrolyte and electrode.^8,10,11^ Nonetheless, nonprecious bifunctional catalysts often suffer from notable drawbacks such as limited stability and intrinsic activity.^12,13^ Hence, the development of catalysts based on Earth-abundant elements with performance comparable to noble-metal-based systems is a crucial task for efficient and durable water electrolysis devices.^14,15^

In recent research, layered triple hydroxides (LTHs) have garnered significant attention owing to their multifarious active sites with notable inherent activity resulting from multimetal coordination, high specific surface area, and favorable charge transfer properties.^16,17^ Among these materials, FeCoNi-based LTHs demonstrate improved catalytic activity due to the synergistic electronic interactions among Fe, Co, and Ni. These interactions facilitate charge transfer and help to stabilize the catalytically active phases during the process of water splitting.^18,19^ For instance, NiCoFe LDH-based composites supported on nickel foam exhibit low overpotentials, reduced Tafel slopes, and excellent durability.^16,20^

Although LTHs exhibit promising activity, they are hindered by low electrical conductivity, which limits electron transport and adversely affects their inherent catalytic efficiency.^21,22^ To address this challenge, heterostructure engineering has emerged as an effective strategy to improve electrocatalytic activity by integrating LTHs with complementary electroactive and conductive components.^23,24^ Such architectures can enhance interfacial charge transfer, increase the availability of active sites, and adjust the adsorption energies of the reaction intermediates. Specifically, combining electroactive transition metal oxides with conductive carbon-based materials fosters synergistic effects by improving active site accessibility, enhancing electron mobility, and facilitating redox processes, thereby significantly boosting overall water splitting performance.^25^

Manganese molybdate (MnMoO_4_) is notable for the presence of multiple oxidation states of its metal ions, which enable tunable redox activity and favorable electrochemical behavior. Its cost-effectiveness, minimal toxicity, availability, and stable performance across a wide pH range make MnMoO_4_ a promising electroactive material for HER and OER applications, either alone or in conjunction with conductive carbon-based additives such as polymers, graphene, and carbon nanotubes. Recent studies on MnMoO_4_-based heterostructures have shown improved electrocatalytic performance through structural and compositional modulation.^26,27^ Nevertheless, the inherent constraints of transition metal oxides require additional improvements in performance. To address this, various carbon-matrix materials have been incorporated into catalytic systems, which are known for their excellent electrical conductivity, high specific surface area, and robust chemical stability, and serve as carriers that facilitate the even distribution of the catalytic active species, mitigating material aggregation.^25,28,29^

Graphene oxide (GO) is one of the most thoroughly studied carbon-based materials due to its sp^2^-hybridized carbon structure, which offers good electrical conductivity, efficient charge transport, and a π-conjugated network advantageous for electrocatalytic reactions.^30^ Recently, GO-based composites have been extensively investigated as versatile electrocatalysts for both HER and OER, where the combination of GO with transition metal compounds has demonstrated notable improvements in catalytic activity and stability.^31–34^ These enhancements are typically ascribed to the synergistic interplay of a high surface area, enhanced electron mobility, and robust interfacial interactions between the GO and electroactive materials.

In this study, an innovative approach is introduced for the first time to produce advanced bifunctional electrocatalysts for water splitting by integrating FeCoNi LTH and MnMoO_4_ nanoparticles as active sites for both the OER and HER, in conjunction with GO on a fluorine tin oxide (FTO) substrate. Although FeCoNi LTH and MnMoO_4_ exhibit favorable electrochemical properties, their inherently low electrical conductivity remains a significant limitation. Therefore, the incorporation of GO as a conductive additive offers a cost-effective strategy to improve the conductivity and electrochemical surface area, attributed to the exceptional mechanical and thermal stability of GO. Moreover, the GO incorporation contributes to the creation of a defect-rich heterostructure, which is advantageous for augmenting the catalytic performance of multimetallic compounds.^35^ To the best of our knowledge, the synthesis of FeCoNi LTH/MnMO_4_/GO heterostructures via a hydrothermal method followed by the electrodeposition of the powder nanocatalysts onto an FTO substrate has not been previously reported. This synthetic strategy is designed to increase the exposure of active sites and improve adhesion to the FTO substrate, thereby enhancing electron transfer efficiency and stability in the system. Overall, the synergistic interaction among FeCoNi LTH, MnMoO_4_, and GO results in a composite with an enhanced density of active sites and accelerated electron transfer dynamics, positioning it as a highly effective bifunctional catalyst for both OER and HER. This work highlights a promising and cost-effective route toward the development of Earth-abundant electrocatalysts with enhanced activity and durability, offering new insights into the rational design and optimization of heterostructured bifunctional electrocatalysts for water splitting applications.

## 2 Experimental Section

### 2.1 Materials and Chemicals

All chemicals and reagents were used as received, including Fe­(NO_3_)_3_·9H_2_O ≥98.0%, Co­(NO_3_)_2_·6H_2_O ≥98.0%, Ni­(NO_3_)_2_·6H_2_O, Mn­(NO_3_)_2_·4H_2_O ≥97.0%, Na_2_MoO_4_·2H_2_O ≥99.0%, Na_2_C_2_O_4_ (sodium oxalate) ≥99.5%, HOCH_2_CH_2_OH (ethylene glycol) ≥99.0%, H_2_SO_4_ ≥98 wt %, NaNO_3_ ≥99.0%, H_2_O_2_ 35 wt %, KMnO_4_ ≥99.0%, and graphite powder (particle size < 45 μm) ≥99.99% from Sigma-Aldrich. All other reagents and solvents employed in this study were of analytical grade, ensuring high purity and reliability for the experimental procedures. Fluorine-doped tin oxide (FTO) glass substrates, exhibiting a resistance of 7 Ω per square, were sourced from Sigma-Aldrich. The FTO substrates (1 × 3.5 cm) were thoroughly cleaned by sequential sonication in soapy water, ethanol, and acetone for 10 min each and finally rinsed with deionized water.

### 2.2 Methods

The structural properties of the synthesized materials were analyzed by X-ray diffraction using graphite monochromatized Cu Kα radiation (λ = 1.54 Å, acceleration voltage of 40 kV and 40 mA, step size of 0.02°, with 2θ ranging from 10 to 60° and step time of 1 s) in the Bruker diffractometer D8 Advance. The average crystallite size (*D*) was determined using the Scherrer equation: *D* = 0.89λ/(βcos θ), where λ represents the X-ray wavelength, β is the full width at half-maximum (fwhm) of the diffraction peaks, and θ is the angle corresponding to the Bragg reflections. The morphological features and elemental composition of the samples were examined by using a field emission scanning electron microscope (FE-SEM) (JEOL JSM 7100F, Japan) at an acceleration voltage of 15 kV and a working distance of 7 mm. Additionally, transmission electron microscopy (TEM) and high-resolution transmission electron microscopy (HR-TEM) analyses were performed utilizing a JEOL JEM 7100F instrument (Japan), complemented by energy-dispersive spectroscopy (EDS) to provide detailed elemental mapping and quantitative analysis. The FTO-electrodeposited material samples were digested in aqua regia at 80 °C for 2 h and analyzed using inductively coupled plasma optical emission spectrometry (ICP-OES) on a Varian 730-ES instrument for the multielement determination. The operating parameters used in ICP-OES were plasma gas flow (12 L min^–1^), nebulizer gas flow (0.4 L min^–1^), auxiliary gas flow (1.0 L min^–1^), radio frequency power (1300 W), pump flow rate (5 rpm, 0.2 mL min^–1^), and radial view. Fourier transform infrared (FTIR) spectroscopy was employed to identify the functional groups present in the samples, utilizing the KBr pellet method on a PerkinElmer FTIR/FIR Frontier (C105496) over the range of 400 to 4000 cm^–1^. X-ray photoelectron spectroscopy (XPS) analyses were performed using a K-Alpha X-ray photoelectron spectrometer from Thermo Scientific to investigate the elemental composition and chemical states of the samples. This technique enabled the detailed assessment of core-level electron binding energies, providing insights into the surface chemistry and oxidation states present within the materials. Raman spectroscopy (Horiba, XploRA plus) (equipped with λ_0_ = 638 nm, 30 mW laser lines) was used to study the vibrational modes of the crystalline lattices.

### 2.3 Preparation of Catalysts

The synthetic process of FeCoNi LTH/MnMoO_4_/GO/FTO is depicted in Scheme 1.

**Scheme 1:**
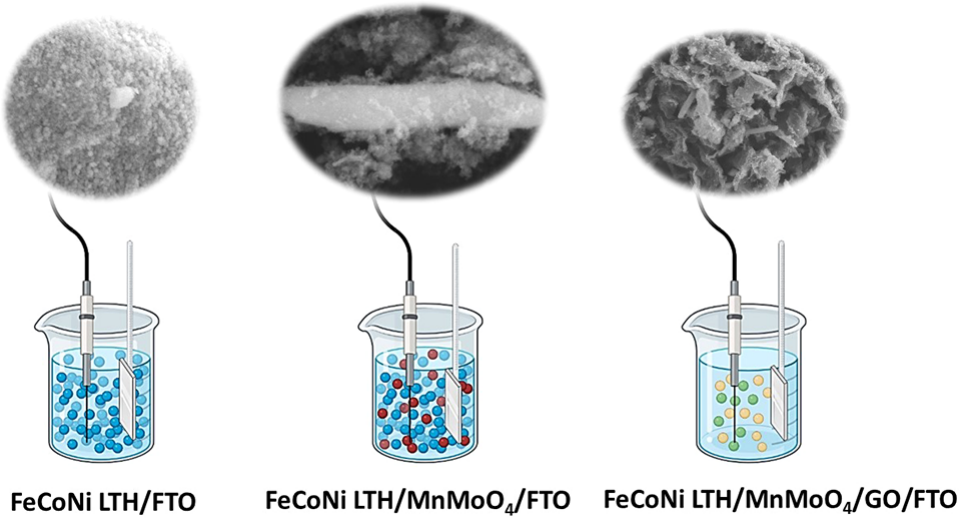
Schematic Illustration the Fabrication of the FeCoNi LTH/MnMoO_4_/GO/FTO Electrocatalyst

#### 2.3.1 Synthesis of FeCoNi LTH Nanoparticles

The FeCoNi LTH was synthesized via a hydrothermal method. Initially, a mixed salt solution was prepared by dissolving 3 mmol of Ni­(NO_3_)_2_·6H_2_O, 2 mmol of Fe­(NO_3_)_3_·9H_2_O, and 2 mmol of Co­(NO_3_)_2_·6H_2_O in 30 mL of deionized water. Concurrently, a secondary solution consisting of 1 mol L^–1^ NaOH and 0.2 mol L^–1^ Na_2_CO_3_ was prepared in an additional 50 mL of deionized water. This solution was gradually introduced into the mixed salt solution under continuous magnetic stirring, with slow dripping at a controlled rate of approximately 1 mL min^–1^ to adjust the final pH of the resulting aqueous mixture to 8.5. The brown colloidal suspension was then transferred to a Teflon-lined stainless-steel autoclave, which was sealed and subjected to a hydrothermal treatment at 150 °C for 20 h, followed by natural cooling to room temperature. The resultant precipitate was collected via centrifugation, thoroughly washed with ultrapure water until a neutral pH was achieved, and subsequently rinsed with ethanol. Finally, the product was dried in a vacuum oven at 60 °C.

#### 2.3.2 Synthesis of MnMoO_4_ Nanorods

MnMoO_4_ nanorods were synthesized by using a straightforward hydrothermal method involving the incorporation of transition metal precursors and sodium oxalate as a complexing agent. Prior to the synthesis, Na_2_MoO_4_·2H_2_O and MnCl_2_·4H_2_O solutions were prepared in a 1:1 molar ratio, with 2 mmol of each salt dissolved separately in 20 mL of deionized water while stirring for 10 min. The Na_2_MoO_4_·2H_2_O solution was then added dropwise to the homogeneous MnCl_2_·4H_2_O solution. Concurrently, 2 mmol of Na_2_C_2_O_4_ and 10 mL of ethylene glycol (EG) were introduced into the mixture with continuous stirring. The resulting solution underwent a hydrothermal treatment at 180 °C for 10 h. The product was collected via centrifugation and extensively washed with deionized water and ethanol to remove impurities. The resulting white precipitate was subsequently dried in an oven at 80 °C for 12 h. Finally, the material was calcined under air at a temperature of 500 °C for 4 h, yielding the final product designated as MnMoO_4_ nanorods.

#### 2.3.3 Preparation of FeCoNi LTH/MnMoO_4_/GO by the Self-Assembled Process

GO was synthesized according to the Hummers method^36^ following established protocols.^37^ Separately, the synthesized graphene powder (20 mg) was dispersed in a solution containing 30 mg of FeCoNi LTH and 30 mg of MnMoO_4_ through ultrasonication for 1 h at 40 kHz and 35 °C. The resulting mixture was then transferred to a Teflon-sealed autoclave and subjected to hydrothermal treatment at 120 °C for 12 h. The resultant materials were washed multiple times with deionized water and ethanol followed by drying at 60 °C to obtain the final product. For comparative analysis, FeCoNi LTH/MnMoO_4_ was synthesized under identical experimental conditions without the addition of GO.

### 2.4 Electrode Preparation

In this experiment, catalyst films were prepared on the FTO by electrodeposition using a chronoamperometric (*i*–*t*) technique within a two-electrode system consisting of a Pt bar as counter electrode and FTO as working electrode (WE), applying a constant potential of −2 V for 1200 s. For the electrolyte preparation, 2 mg of catalyst, 10 mL of water, and 0.6 mg each of boric acid and KI were mixed. The mixture underwent ultrasonication for 1 h at 40 kHz and 35 °C prior to the deposition process. The electrodes were oriented vertically and submerged in a 10 mL suspension within a glass beaker. Following the electrodeposition at room temperature (25 ± 2 °C) under ambient conditions, the electrodes were rigorously rinsed with deionized water to eliminate any residual or loosely bound particles, air-dried, and stored in a desiccator for subsequent applications. The resulting modified electrodes were designated as FeCoNi LTH/FTO, FeCoNi LTH/MnMoO_4_/FTO, and FeCoNi LTH/MnMoO_4_/GO/FTO. All electrodes were prepared at least two times under identical conditions, showing good reproducibility with deviations within an acceptable experimental error. The catalyst mass loadings were assessed by the difference of the weight of the FTO electrodes before and after digestion for the ICP-OES analysis of the deposited films. The mass loadings were roughly 0.28, 0.46, and 0.63 mg cm^–2^ for FeCoNi LTH/FTO, FeCoNi LTH/MnMoO_4_/FTO, and FeCoNi LTH/MnMoO_4_/GO/FTO, respectively.

Ru was first electrodeposited onto a cleaned FTO substrate by using a three-electrode configuration with Hg/Hg_2_Cl_2_ 1 mol L^–1^ KCl (saturated calomel electrode, SCE) as the reference electrode and a Pt foil as the counter electrode. The electrolyte contained 5 mM RuCl_3_ and 0.1 M KCl. Deposition was conducted potentiostatically at −0.20 V vs SCE for 600 s. The deposited electrode was subsequently annealed in air at 350 °C for 2 h to obtain RuO_2_.

Pt was electrodeposited onto a precleaned FTO substrate (geometric area: 1 cm^2^) using a standard three-electrode configuration with FTO as the working electrode, a Pt foil as the counter electrode, and SCE as the reference electrode. The deposition electrolyte consisted of 5 mM H_2_PtCl_6_ dissolved in 0.5 M H_2_SO_4_. Electrodeposition was performed potentiostatically at −0.20 V vs SCE for 600 s. After deposition, the electrode was thoroughly rinsed with deionized water and dried under ambient conditions.

### 2.5 Electrochemical Tests

The electrode preparation and electrochemical characterization encompassing cyclic voltammetry (CV), electrochemical impedance spectroscopy (EIS), and linear sweep voltammetry (LSV) were conducted using an Autolab PGSTAT302N potentiostat/galvanostat (Metrohm, Herisau, Switzerland) operated via the NOVA software version 2.1.5. The experiments employed a standard three-electrode configuration comprising FTO glass substrates modified with the synthesized nanocatalysts as WE with an active area of 1 cm^2^, a platinum bar as the counter electrode, and SCE as the reference. For these measurements, 35 mL of a 1 mol L^–1^ KOH solution was used as the electrolyte. All potentials were converted to the reversible hydrogen electrode (RHE) scale by eq 1:

$$E_{\mathrm{RHE}}=E_{\mathrm{SCE}}+0.244\;V+0.059\;V\times \mathrm{pH}$$ 1

The electrocatalytic performance of the catalysts for OER and HER was evaluated using LSV at a scan rate of 5 mV s^–1^. Prior to the LSV measurements, the catalysts underwent electrochemical activation through CV at a scan rate of 50 mV s^–1^ until a stable cyclic voltammogram was achieved.

The Tafel slope was calculated by eq 2:

$$b=\frac{\partial \eta }{\partial \log\log\left(i\right)}=\frac{2.303RT}{\alpha F}$$ 2

where *b* denotes the Tafel slope in mV dec^–1^, η represents the overpotential measured in V, *R* is the universal gas constant, *T* is the absolute temperature in kelvin, *F* corresponds to the Faraday constant, and α is the anodic transfer coefficient.

The electrochemically active surface area (ECSA) was assessed from the electrochemical double layer capacitance (*C*_dl_) of the electrodes by the CV method measured in a non-Faradaic region, with scan rates ranging from 0.05 to 0.3 V s^–1^. The capacitive current (*i*_C_) (*i*_C_ = *C*_dl_ × ν) was obtained from both anodic and cathodic responses, while *C*_dl_ was determined from the average of the slopes of the anodic and cathodic linear plots of *i*_C_ against the scan rate (ν). The ECSA was determined by eq 3:

$$\mathrm{ECSA}=C_{\mathrm{dl}}/C_{s}$$ 3

where *C*_s_ denotes the specific capacitance of the material in 1 mol L^–1^ KOH, which is reported to be 0.040 mF cm^–2^.^38^

To ensure a reliable comparison of electrochemical activities among different catalysts, automatic *i*R compensation was applied to all polarization curves, where *R* represents the internal resistance of the solution. The electrode and solution resistances were characterized using EIS, conducted over a frequency range of 10^5^ to 0.01 Hz with an AC voltage amplitude of 0.01 V_RMS_ at an OER and HER overpotential. Notably, no *i*R compensation was applied during these EIS measurements. The charge transfer resistance (*R*_ct_) and solution resistance (*R*_s_) were extracted through electrochemical fitting based on a Randles equivalent circuit model, incorporating constant phase elements (CPE) and Warburg elements (W) to account for nonideal behavior.

The specific activity was calculated by dividing the current at η = 300 mV for the OER and at η = 200 mV for the HER by the ECSA (cm^2^). This calculation can be expressed by eq 4:

SA=i/ECSA 4

The mass activity (MA) was calculated by dividing the current density at η = 300 mV for the OER and at η = 200 mV for the HER by the catalyst loaded at the electrode according to eq 5:

$$\mathrm{MA}\;[A\;g^{-1}]=j\;[A\;{\mathrm{cm}}^{-2}]/m\;[g\;{\mathrm{cm}}^{-2}]$$ 5

The turnover frequency (TOF) was calculated from LSV curves at selected potentials according to eq 6.

$$\mathrm{TOF}=\frac{\left|i\right|}{nFN_{\mathrm{active}}}$$ 6

where *i* is the measured current (A) at a given overpotential, *F* is the Faraday constant (96,485 C mol^–1^), *n* is the number of electrons involved in the reaction (*n* = 4 for OER and *n* = 2 for HER), and *N*_active_ is the molar number of active sites. *N*_active_ (eq 7) was estimated from the total metal loading determined by the ICP-OES analysis of the catalysts.

$$N_{\mathrm{active}}=\frac{i^{m}}{i^{M}}\sum_{i}$$ 7

where *i* ^m^ is the mass of metal *i* determined by ICP-OES (g) and *i* ^M^ is the molar mass of metal *i* (g mol^–1^).

Stability tests for the OER and HER were conducted by chronoamperometry at 10 and −10 mA cm^–2^ for 18 h.

The overall water splitting test was conducted in a two-electrode system with the same catalyst at both FTO electrodes. LSV was measured at the scan rate of 5.0 mV s^–1^, and the long-term stability *i*–*t* curve was measured by chronoamperometry at 1.57 V for 31 h.

All electrochemical measurements were carried out in duplicates.

## 3 Results and Discussion

### 3.1 Characterization of the Catalysts

The crystalline structures of the as-synthesized samples, FeCoNi LTH, MnMoO_4_, FeCoNi LTH/MnMoO_4_, and FeCoNi LTH/MnMoO_4_/GO, were characterized by XRD as shown in Figure 1. The XRD pattern of FeCoNi LTH shows peaks at 2θ of 11.6°, 18.41°, 23.4°, 30.75°, 35.71°, 43.56°, 53.91°, and 57.37°, which match well with the (003), (111), (006), (220), (311), (400), (422), and (511) planes of LTH, respectively, in agreement with JCPDS No. 01-074-2081. Regarding the diffractogram of MnMoO_4_, the analysis reveals distinct peaks at 12.90°, 18.80°, 22.83°, 24.80°, 25.78°, 25.92°, 26.63°, 26.98°, 27.70°, 31.28°, 32.10°, 32.97°, 35.64°, 37.72°, 39.01°, 40.38°, 42.78°, 43.99°, 45.73°, 51.11°, 52.2°, 53°, 54.38°, and 57.33°, which correspond to the crystallographic planes of the monoclinic crystal system, specifically (110), (020), (021), (201), (220), (002), (−112), (−202), (−311), (112), (022), (−222), (400), (040), (330), (222), (−422), (113), (241), (−204), (−531), (440), (422), and (620), as referenced in JCPDS No. 01-082-2166. Notably, the absence of any additional impurity phases in the diffraction pattern reinforces the high purity. In the XRD pattern of FeCoNi LTH/MnMoO_4_, all the main peaks of FeCoNi LTH and MnMoO_4_ are well preserved. GO exhibits two prominent diffraction peaks at 11.6° and 42.9°, which correspond to the (002) and (100) lattice planes, respectively. The XRD pattern of the FeCoNi LTH/MnMoO_4_/GO nanocatalyst reveals that the primary diffraction peaks associated with the FeCoNi LTH/MnMoO_4_ sample retain their positions, albeit with decreased intensity upon the incorporation of GO. Notably, no additional significant diffraction peaks are detected, suggesting that the introduction of GO does not induce substantial structural distortion. The observed decrease in peak intensity can be attributed to the fine dispersion of the nanocatalyst matrix within GO. Using Scherrer’s equation, the crystallite sizes of the prepared samples were estimated, resulting in values of 15.1 nm for FeCoNi LTH and 74.1 nm for MnMoO_4_, which were slightly decreased in the composites: FeCoNi LTH/MnMoO_4_ (13.4 nm for FeCoNi LTH and 73.6 nm for MnMoO_4_) and FeCoNi LTH/MnMoO_4_/GO (11 nm for FeCoNi LTH and 69.4 nm for MnMoO_4_). These results provide compelling evidence for the successful fabrication of the catalysts.

**Figure 1:**
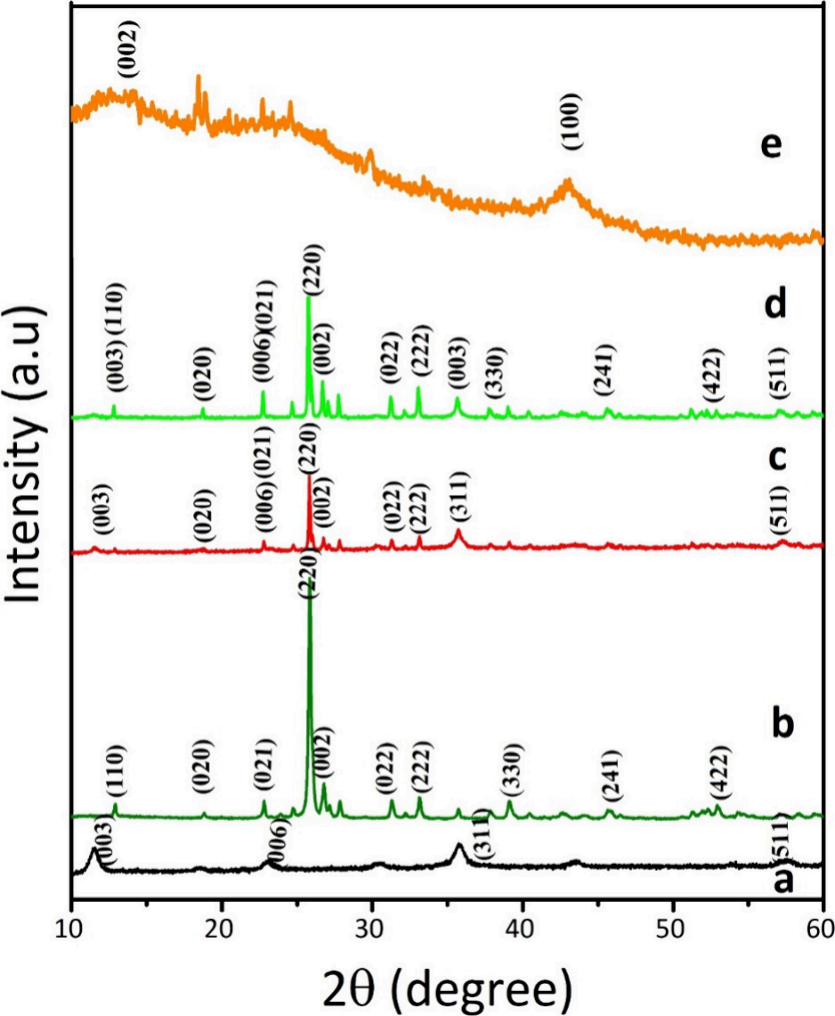
XRD patterns of (a) FeCoNi LTH, (b) MnMoO_4_, (c) FeCoNi LTH/MnMoO_4_, (d) FeCoNi LTH/MnMoO_4_/GO, and (e) GO.

The morphology of the electrocatalysts was examined using FESEM, as presented in Figure 2. The FeCoNi LTH (Figure 2a) comprises spherical nanoparticles (NPs) that demonstrate a significant tendency to agglomerate while maintaining a nearly homogeneous particle size distribution. Following the integration of the well-defined MnMoO_4_ nanorods (NRs) (Figure 2b), the image of the composite FeCoNi LTH/MnMoO_4_ (Figure 2c) shows that numerous FeCoNi LTH particles exhibiting nanoscale morphological irregularities are dispersed unevenly across the surface of the MnMoO_4_ hosts. This arrangement reflects a lack of an orderly distribution in their spatial configuration. The FESEM examination of GO demonstrates the presence of two-dimensional sheets featuring a distinctly thin and wrinkled morphology, as illustrated in Figure 2d. This morphology showcases distinct layering and irregular crumpling, indicative of the coexistence of both multilayered and few-layered GO. It is evident that the ultrafine FeCoNi LTH NPs and MnMoO_4_ NRs are densely anchored onto the GO sheets with minimal aggregation in FeCoNi LTH/MnMoO_4_/GO (Figure 2e). The smooth texture of the GO sheets is now completely covered with irregularly distributed nanoparticles and nanorods. Notably, the incorporation of GO did not alter significantly the inherent morphology of the individual components, allowing each material to remain distinct within the final nanocatalyst structure.

**Figure 2:**
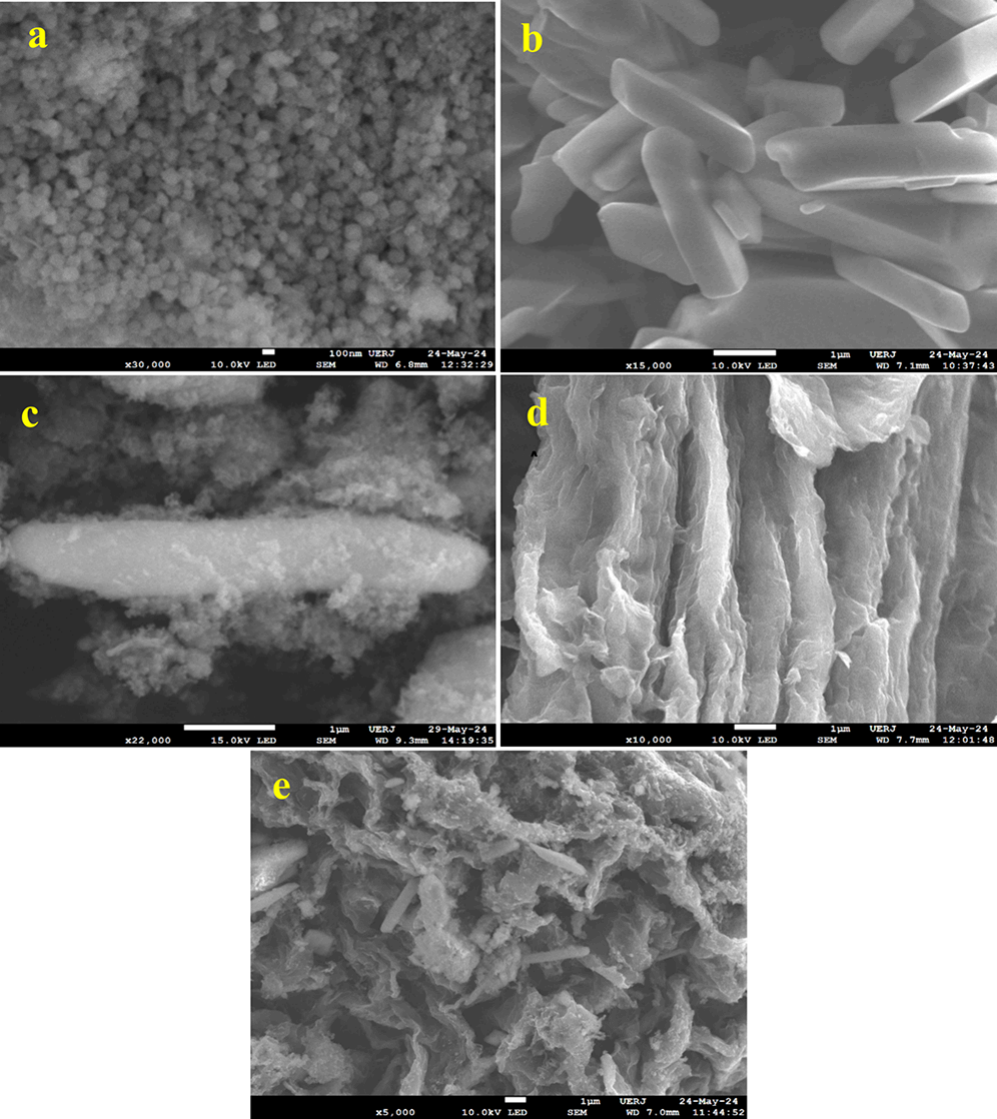
FESEM images of (a) FeCoNi LTH, (b) MnMoO_4_, (c) FeCoNi LTH/MnMoO_4_, (d) GO, and (e) FeCoNi LTH/MnMoO_4_/GO.

Figure 3 presents the TEM and HRTEM images and elemental mapping, providing detailed insight into the morphology and chemical composition of the FeCoNi LTH/MnMoO_4_/GO catalyst. The images with different magnifications shown in Figure 3a,b reveal three distinct morphologies in the prepared sample: wrinkled sheets, which are characteristic of GO; well-separated smaller NPs; and connected bigger NPs. The SEM results already showed that the smaller nanoparticles are FeCoNi LTH and the connected bigger ones are MnMoO_4_, and this conclusion was confirmed by the EDS elemental mapping results shown in Figure 3e. These spherical FeCoNi LTH NPs (with a particle size around 11 nm) consist of numerous smaller subunits densely clustered together and are securely anchored onto GO. MnMoO_4_ NRs have an average particle size of around 42 nm. Both FeCoNi LTH NPs and MnMoO_4_ NRs retain their structural integrity and distinct morphologies on the GO surface. The NPs exhibit minimal agglomeration, forming well-defined spherical or rod-like structures. The HRTEM image presented in Figure 3c reveals well-aligned lattice stripes, indicating the high crystallinity of the NPs. The measured internal lattice fringe spacings of 0.23 and 0.26 nm in the lower left and right regions are attributed to the (400) and (311) crystal planes of FeCoNi LTH. Meanwhile, the lattice spacings of 0.43 and 0.47 nm observed in the up and down regions correspond to the (201) and (020) planes of MnMoO_4_, as well as the (002) plane of graphene oxide (GO) with a lattice spacing of 0.31 nm.^39^ These observations are consistent with the significant peaks in the XRD patterns of FeCoNi LTH, MnMoO_4_, and GO, aligned with the (111), (102), and (002) planes, respectively. The integrated interface among the three domains demonstrates the robust anchoring of FeCoNi LTH and MnMoO_4_ onto the surface of the GO nanosheets. This strong interfacial interaction potentially leads to pronounced synergistic effects among the materials, thereby enhancing electron transport and catalytic stability. Additionally, the crystallinity and phase characteristics of the materials were corroborated by using selected area electron diffraction (SAED) analysis. The SAED pattern (Figure 3d) for the FeCoNi LTH/MnMoO_4_/GO composite exhibits distinct diffraction spots and concentric rings corresponding to the (003), (110), (111), (220), (222), (−511), (114), and (062) crystal planes. The results obtained are in complete agreement with the XRD analysis, further validating the structural composition. Furthermore, the EDS data of the FeCoNi LTH/MnMoO_4_/GO catalyst (Figure S1) reveal the presence of the six elements, i.e., Fe, Co, Ni, Mn, Mo, and C, confirming the successful synthesis and integration of the FeCoNi LTH/MnMoO_4_/GO catalyst.

**Figure 3:**
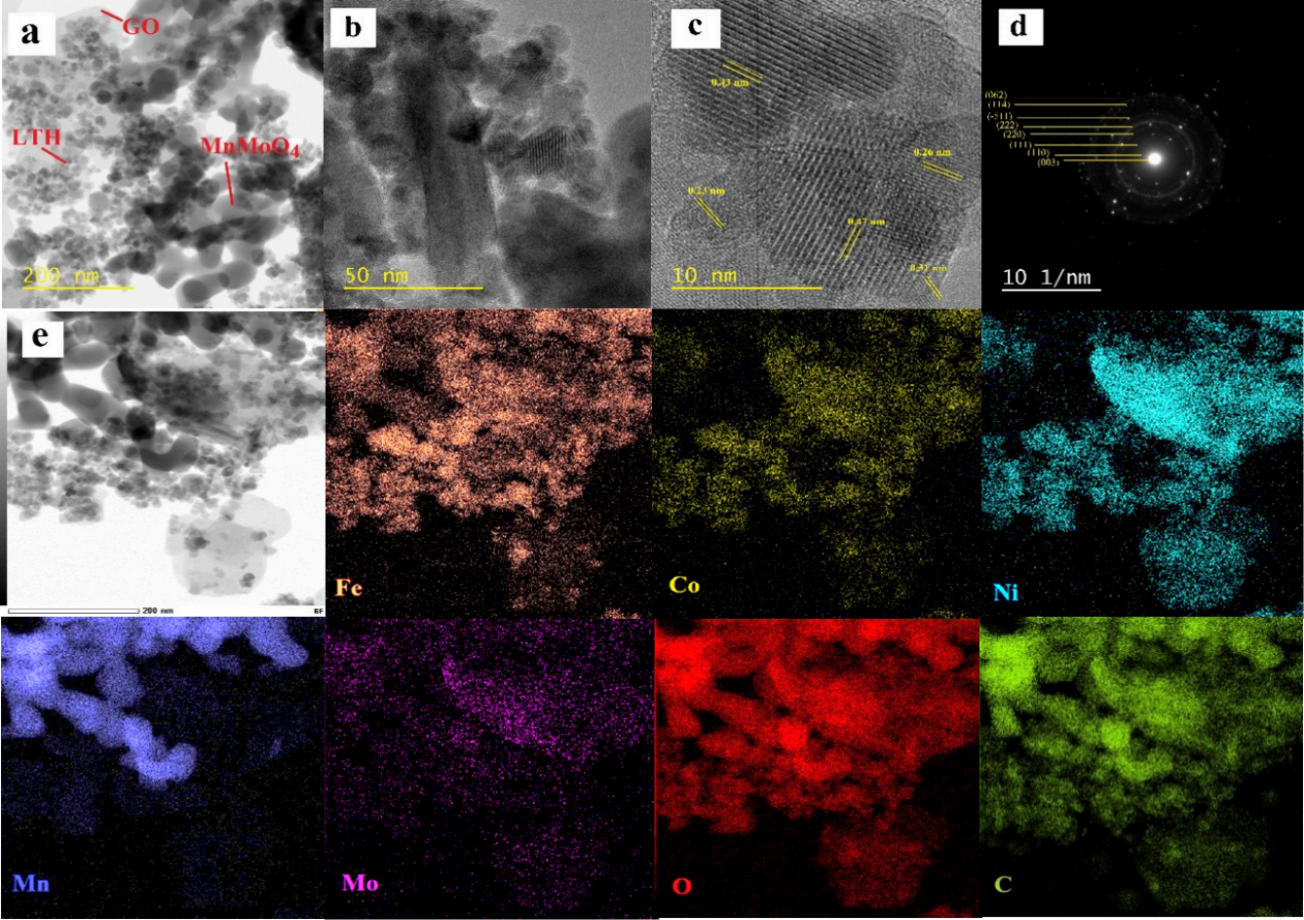
(a, b) TEM and (c) HRTEM images, (d) SAED pattern, and (e) bright-field scanning TEM image and corresponding elemental mappings of FeCoNi LTH/MnMoO_4_/GO.

FTIR spectroscopy was employed to analyze the structural characteristics of the synthesized nanocatalysts across the spectral range of 400–4000 cm^–1^ (Figure 4). The characteristic absorption bands observed near 3440 and 1631 cm^–1^ in all samples correspond to the stretching vibrations of structural hydroxyl (OH) groups and the bending vibrations (δHOH) of adsorbed water molecules, respectively.^19^ The observed weak shoulder at 2933 cm^–1^ is likely indicative of the hydroxyl (OH) stretching vibrational mode associated with water molecules. The absorption band observed near 1386 cm^–1^ corresponds to the stretching vibrations of the N–O bonds in the nitrate (NO_3_) groups. Additionally, the bands observed below 1000 cm^–1^ correspond to various metal–oxygen skeletal vibrations including M–O, M–O–M, and M–OH interactions. Notably, the pronounced peak at 607 cm^–1^ in the FeCoNi LTH sample suggests a higher occupancy of Fe^2+^ ions at the tetrahedral coordination sites, potentially leading to an increased Fe^2+^ to Fe^3+^ ratio.^40,41^ The FTIR spectrum of MnMoO_4_ exhibits four prominent peaks at 720, 798, 863, and 945 cm^–1^, confirming the metal–oxygen bonding characteristic of the MnMoO_4_ crystalline framework. These spectral features are indicative of the α phase of MnMoO_4_ characterized by tetrahedral coordination of the central molybdenum atom at the surface.^42,43^ The spectral peaks observed at 720 and 798 cm^–1^ correspond to the stretching vibrations of Mo–O bonds within tetrahedral Mo–O_4_ units and indicate the presence of metal–metal bonding interactions.^43^ Additionally, the peak at 945 cm^–1^ is indicative of stretching vibrations associated with the Mo=O moiety characteristic of the MoO_3_ phase, while the band at 863 cm^–1^ is associated with Mo–O–Mo bending vibrations.^42,44^ These pronounced bands confirm the tetrahedral coordination of oxygen around the Mo atoms in the MnMoO_4_ unit cell. The FTIR spectrum of the FeCoNi LTH/MnMoO_4_ nanocatalysts displays a merging of characteristic bands from both pure FeCoNi LTH and MnMoO_4_ despite some overlapping vibrational modes. These findings strongly support the successful synthesis of the FeCoNi LTH/MnMoO_4_ composite. The FTIR spectrum of GO displays a prominent peak at 1723 cm^–1^, which is associated with C=O stretching vibrations from carboxylic acid (−COOH) groups and carbonyl functionalities. Additionally, the significant peaks observed at 3429 and 1626 cm^–1^ correspond to the stretching vibrations of the hydroxyl (−OH) groups and the C=C bending modes, respectively. Peaks observed at 1383 cm^–1^ and in the range of 2923–2853 cm^–1^ are attributed to C–OH stretching of carboxylic acid groups and the symmetric and asymmetric C–H stretching modes of aromatic rings. The band at 1070 cm^–1^ represents the stretching vibration of the alkoxy (C–O) group.^37^ Notably, following the interaction of the FeCoNi LTH/MnMoO_4_ nanocatalysts with GO, a slight red shift of these bands to lower wavenumbers was observed, indicative of covalent bonding between the nanocatalysts and the GO surface.

**Figure 4:**
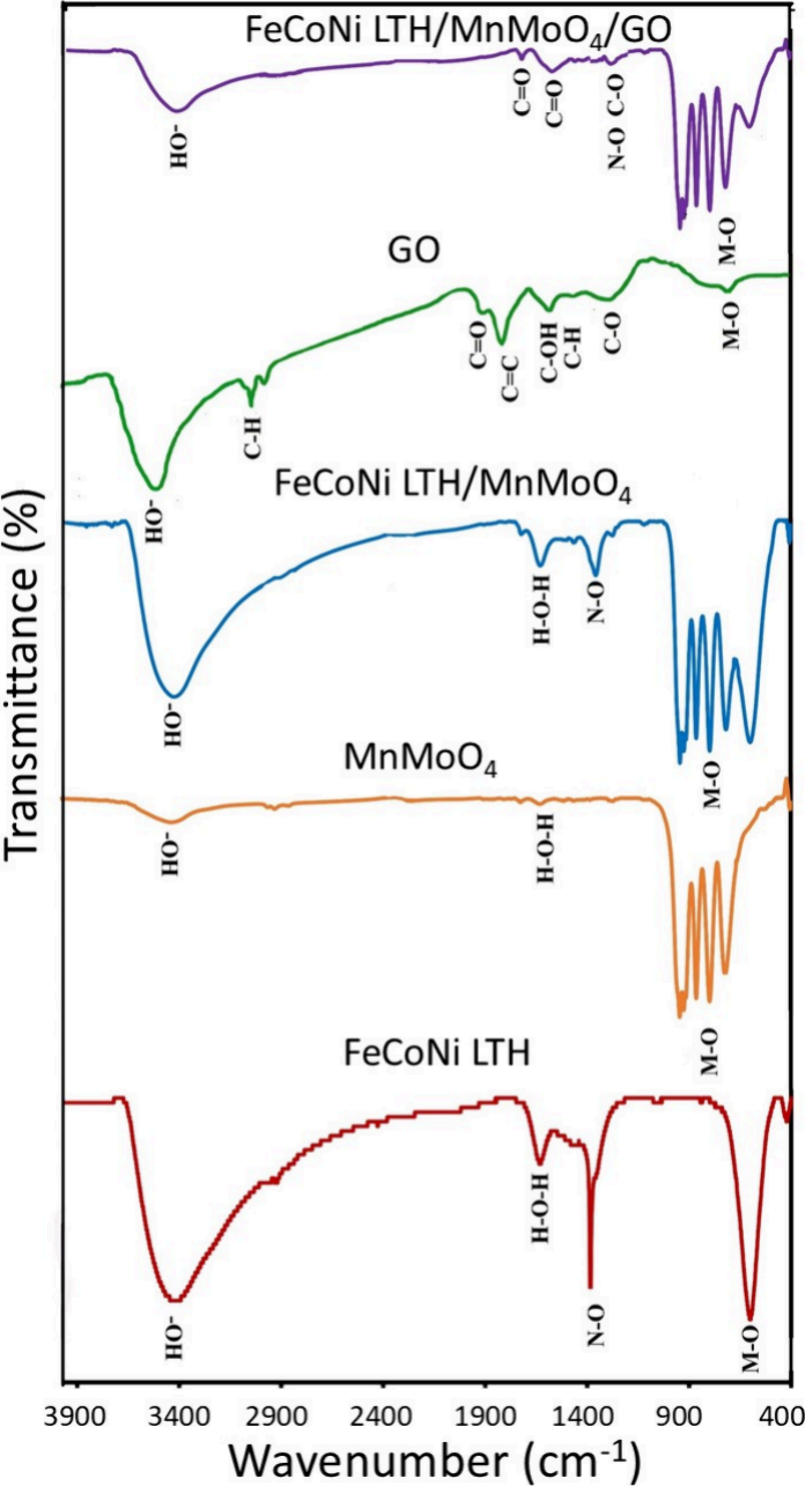
FTIR spectra of the nanocatalysts.

To further investigate the valence states of elements and the surface chemical composition of the FeCoNi LTH/MnMoO_4_/GO nanocatalyst, we performed XPS analysis. The detailed XPS survey spectrum (Figure 5a) reveals the presence of C 1s, O 1s, Co 2p, Ni 2p, Fe 2p, Mn 3d, and Mo 3d, confirming the elemental makeup of the FeCoNi LTH/MnMoO_4_/GO nanocatalyst. The quantitative XPS fitting parameters (binding energy, fwhm, and atomic percentages) have been extracted and are provided in Tables S1–S3. From the survey spectrum, carbon (32.4 at. %) and oxygen (47.6 at. %) are the major elements, although nickel (2.3 at. %), iron (3.3 at. %), cobalt (3.5 at. %), and molybdenum (2.3 at. %) are in close quantities at the surface of the catalysts. However, an enrichment of manganese (8.6 at. %) at the surface was observed. In the survey spectrum, transitions corresponding to C 1s and O 1s are identified at binding energies of 286.6 and 533.7 eV, respectively. Figure 5b–h displays the high-resolution XPS spectra for the C 1s, O 1s, Ni 2p, Co 2p, Fe 2p, Mn 3d, and Mo 3d regions, respectively, providing detailed insights into the electronic states of these elements. The C 1s spectrum of FeCoNi LTH/MnMoO_4_/GO (Figure 5b) reveals a complex deconvolution comprising four distinct peaks at binding energies of 284.8, 285.9, 286.7, and 288.9 eV. These peaks correspond to various carbon species, including aromatic carbon atoms (C=C), aliphatic carbon (C–C), as well as functional groups such as hydroxy C–OH and carboxylic (C=O) moieties.^33,45,46^ The chemical shifts observed in this study arise from the differences in the binding energy (BE) of electrons associated with specific atomic chemical states compared to those of the corresponding pure elements. Notably, these values are consistent with those documented in the previous literature for GO, confirming their reliability.^47^ The considerable contribution of oxygen-containing functional groups confirms the effective integration of GO. In the O 1s spectrum (Figure 5c), peaks appear at 529.6, 530.6, 531.9, and 533 eV, corresponding to surface lattice oxygen, oxygen vacancies, chemisorbed oxygen, and oxygen in C=O, C–OH, and C–O groups, respectively. These O 1s signals also reflect different chemical environments, attributed to M–O, M–OH, and adsorbed water species.^46^ Significantly, the existence of oxygen vacancies suggests a defect-rich surface that enhances catalytic performance by improving electronic conductivity, strengthening the adsorption of reaction intermediates, and facilitating faster charge transfer during OER and HER. In the Ni 2p spectral region (Figure 5d), peaks located at approximately 855.6 and 857.8 eV correspond to Ni^2+^ and Ni^3+^ states in the 2p_3/2_ region, while those near 873.5 and 874.2 eV represent the Ni^2+^ and Ni^3+^ states in the 2p_1_/_2_ region. From the Ni 2p_3/2_ region, the Ni^2+^/Ni^3+^ molar ratio was calculated as 1.76. Additionally, two satellite peaks emerge at about 861.4 and 879.8 eV. Notably, the simultaneous occurrence of Ni^2+^ and Ni^3+^ oxidation states is frequently documented in the characterization of nickel oxides.^48,49^ As depicted in Figure 5e, the spectral features at approximately 705.7, 711.1, and 712.7 eV are assigned to the 2p_3/2_ states of iron in its Fe^0^, Fe^2+^, and Fe^3+^ oxidation states, respectively, in an Fe^2+^/Fe^3+^ molar ratio of 1.47. In contrast, the peaks located at around 719.2, 723.8, and 726.2 eV correspond to the 2p_1/2_ states of the same iron oxidation states. Additionally, satellite peaks are also observed at 716.4 and 732.9 eV, providing insights into the electron correlation effects and the electronic structure of iron within this system.^49,50^ Figure 5f presents three distinct peaks positioned at approximately 775.9, 780.8, and 784.7 eV, which are indicative of the 2p_3/2_ electronic states of cobalt in its Co^0^, Co^2+^, and Co^3+^ forms, respectively, in a Co^2+^/Co^3+^ molar ratio of 0.72. Furthermore, another set of peaks observed at around 795.8, 796.6, and 798.4 eV corresponds to the 2p_1/2_ states of the same cobalt oxidation states. Typical of cobalt oxides, shakeup satellite peaks are noted at 786.2 and 803.7 eV.^51^ The presence of mixed-valence states (Ni^2+^/Ni^3+^, Co^2+^/Co^3+^, and Fe^2+^/Fe^3+^) indicates significant electronic interactions within the heterostructure, offering flexible redox centers that promote electron transfer and stabilize reaction intermediates throughout the process of water splitting, with a prevalence on Ni^2+^, Co^3+^, and Fe^2+^ at the surface of the catalyst. Figure 4g,h illustrates the Mn 2p and Mo 3d spectra for α-MnMoO_4_ within the FeCoNi LTH/MnMoO_4_/GO nanocatalysts. In the Mn 2p spectrum (Figure 5g), four prominent peaks are detected at 642.2, 643.40, 653.8, and 655.37 eV, corresponding to the Mn 2p_3/2_ and Mn 2p_1/2_ energy states, with an 11.6 eV separation, characteristic of Mn^3+^ and Mn^4+^ ions in MnMoO_4_. The Mn^3+^/Mn^4+^ molar ratio calculated from the Mn 2p_3/2_ peak is 0.57. In the Mo 3d XPS spectrum (Figure 5h), two prominent peaks appear at 232.1 and 235.2 eV, which are attributed to the Mo 3d_5/2_ and Mo 3d_3/2_ levels of the Mo^6+^ ions, respectively. This confirms that molybdenum within the particles is present only in the Mo^6+^ oxidation state.^28^ The presence of high-valence species promotes enhanced electrooxidation reactions, contributing to an improved water-splitting performance. The presence of Mn^3+^ and Mn^4+^ with a prevalence of the Mn^4+^ valence state at the surface of the catalyst, when the expected from the stoichiometry of MnMoO_4_ is Mn^2+^, indicates that Mn offers highly oxidizing redox-active sites while Mo improves electronic conductivity, working together to enhance the catalytic kinetics. Overall, quantitative XPS analysis validates the development of a defect-rich, multivalent heterostructure characterized by a significant presence of oxygen vacancies and electronically interconnected interfaces. These attributes collectively account for the enhanced intrinsic activity, increased turnover frequency, and improved stability observed in the electrochemical HER and the OER performance discussed below.

**Figure 5:**
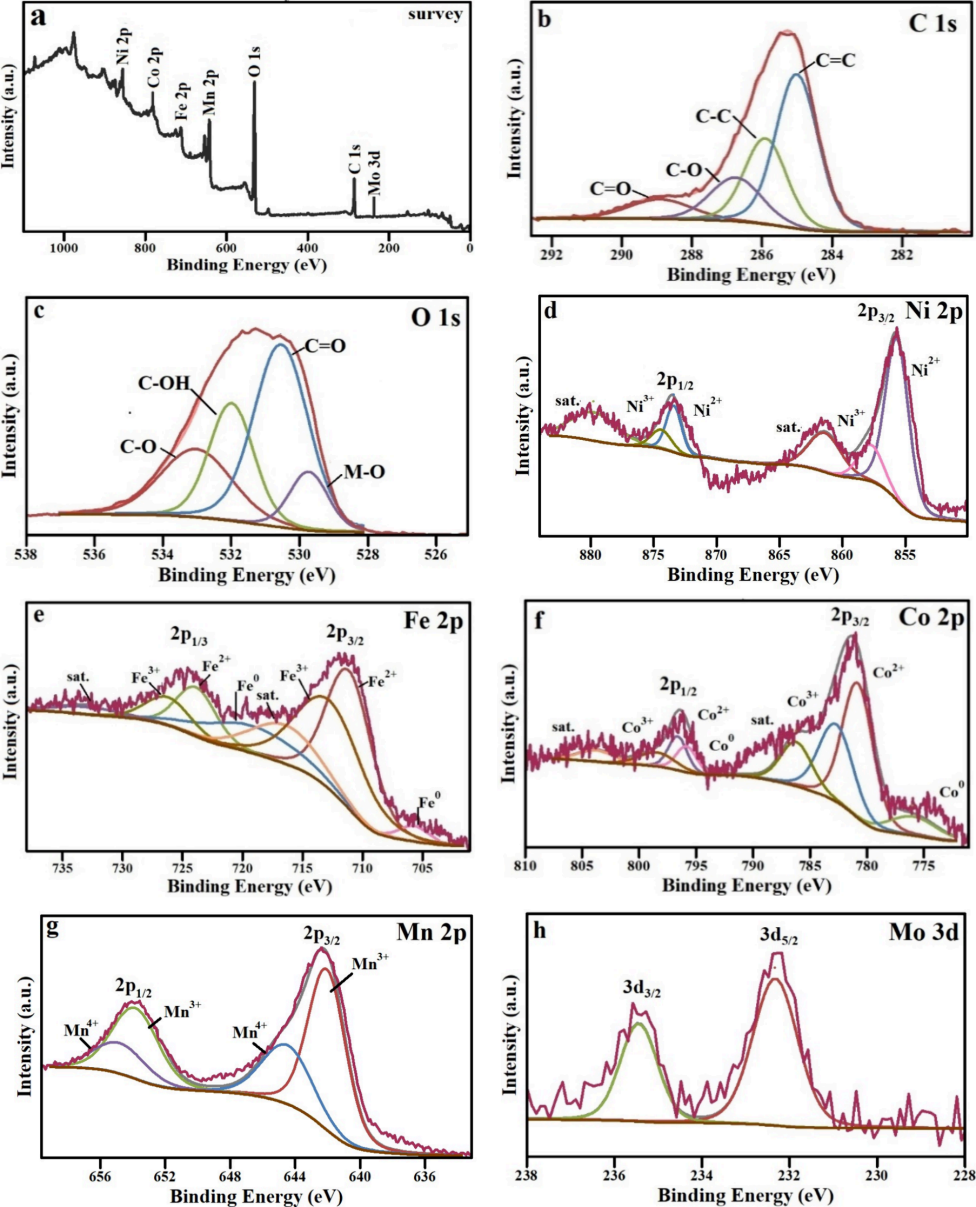
(a) XPS survey scan spectrum of the FeCoNi LTH/MnMoO_4_/GO nanocatalyst. High-resolution XPS spectra of (b) C 1s, (c) O 1s, (d) Ni 2p, (e) Fe 2p, (f) Co 2p, (g) Mn 2p, and (h) Mo 3d.

Raman spectroscopy is an important tool for investigating the crystallinity, comprehending the nature of bonding, and assessing defect states in nanocrystalline materials.^52^ The vibrational properties and structural interactions of FeCoNi LTH, MnMoO_4_, GO, and their composites were examined by analyzing their Raman spectra (Figure 6). As shown in the Raman spectrum of FeCoNi LTH, the prominent peaks below 1000 cm^–1^, with a strong feature around 600 cm^–1^ and a broad band ranging from 200 to 700 cm^–1^, are related to the M–O and M–OH bonds (where M = Co, Ni, Fe), corresponding to the LTH structure.^53^ The presence of nitrate ions can be confirmed by the peak observed at 1300 cm^–1^.^54^ According to the literature, α-MnMoO_4_ nanoparticles exhibit 13 Raman-active vibrational modes (3A_g_ + 5B_g_ + 5E_g_) as determined by group theory.^55^ It is clear that the high intense peak identified at 937 cm^–1^ belongs to the A_g_ mode, resulting from symmetrical vibration of Mo(1)­O(2) (Mo=O).^25^ The subsequent significant bands appearing at 874 and 812 cm^–1^ are likely associated with the Mo(1)­O(1) symmetric stretching vibration (O–Mo–O), corresponding to A_g_ (+B_g_) and B_g_ (+A_g_) modes.^56^ Furthermore, an additional high peak at 645 cm^–1^ can be assigned to the A_g_ mode and coupled vibrations between MoO_4_ units and MnO_6_ octahedra that represent the connectivity in the lattice. The Mo–O stretching modes concerning bridging oxygen atoms (e.g., Mo–O–Mo or Mo–O–Mn interactions) can be included in this region. The lower-frequency bands at 344 and 267 cm^–1^ may be related to the presence of the A_g_^b^ (+B_g_) vibration of the Mo–O group. Hence, the Raman spectra distinctly demonstrate the formation of MnMoO_4_ nanoparticles without any defects or crystalline phase change.^57^ The GO spectrum indicates distinctive D and G bands at approximately ∼1341 and ∼1600 cm^–1^, respectively. The G band (the E_2g_ mode) is responsible for the in-plane vibration, which corresponds to ordered sp^2^-bonded carbon atoms, while the D band, linked to the A_1g_ symmetry mode, indicates the presence of edges, various defects, and disordered carbon resulting from the vibrations of sp^3^-bonded carbon atoms.^58^ The intensity ratio of the D band to the G band (ID/IG = 1.08) is an indication of high disorder that is typical in GO, derived from defects associated with vacancies, grain boundaries, and amorphous carbon structures. The spectrum of the FeCoNi LTH/MnMoO_4_ composite shows features of both components, with peaks below 600 cm^–1^ similar to the ones of FeCoNi LTH and a distinct peak around 900 cm^–1^ corresponding to the Mo–O stretching mode of MnMoO_4_, thereby confirming the successful formation of the composite. Nevertheless, the reduced intensity of MnMoO_4_ peaks in comparison to that of its pure form could be evidence of the potential interactions or dilution effects within the composite structure. The spectrum of FeCoNi LTH/MnMoO_4_/GO also contains the G and D bands of GO at ∼1603 and ∼1350 cm^–1^ while maintaining the M–O vibrations of FeCoNi LTH below 600 cm^–1^ and the Mo–O peak of MnMoO_4_ around 900 cm^–1^. In addition, in this composite, the red shift of the MnMoO_4_ and FeCoNi LTH bands indicates that the GO does not only act as a basal plane for the crystalline MnMoO_4_ and FeCoNi LTH but rather forms some sort of chemical interactions among them. The presence of all characteristic peaks ensures that the individual components in the composites have preserved their structural integrity. The unchanged D/G ratio of GO in the ternary composite alongside the observed reduction in MnMoO_4_ and FeCoNi LTH peak intensity suggests surface adsorption or coordination-induced interactions, therefore indicating minimal alteration to the defect structure of GO. The results suggest that the interaction of the GO with FeCoNi LTH/MnMoO_4_ can be both chemical covalent bonding at the oxygen-containing sites as well as van der Waals force interactions with GO conjugated domains.

**Figure 6:**
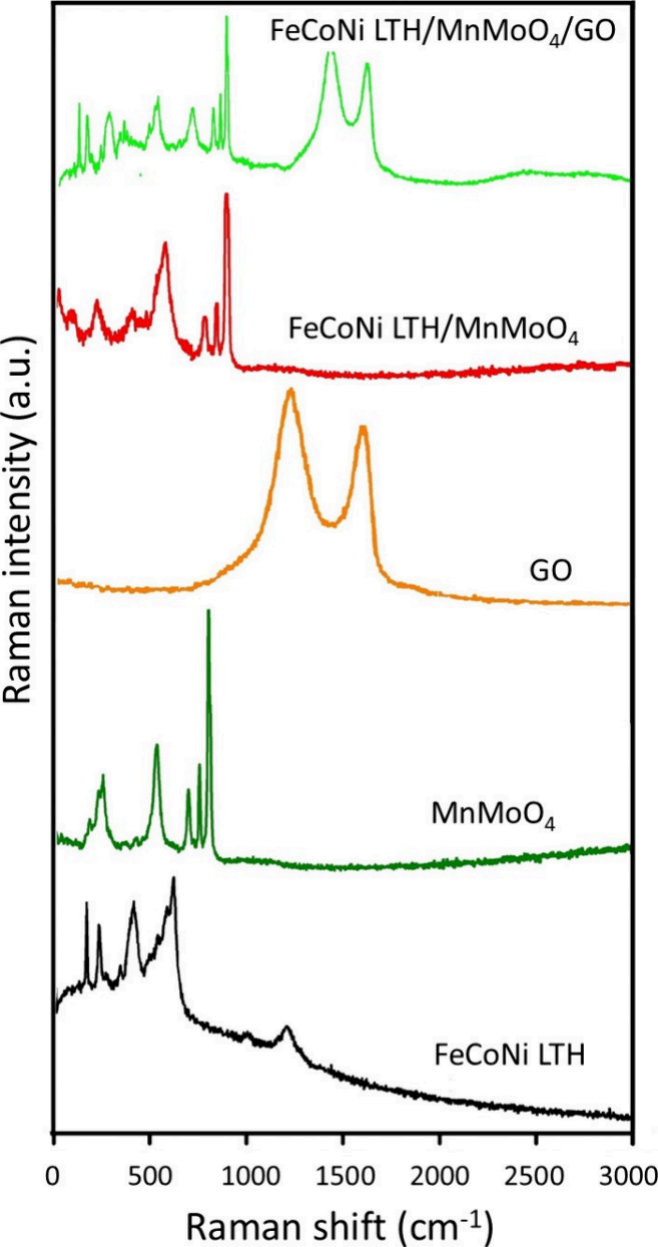
Raman spectra of the nanocatalysts.

### 3.2 Water Splitting Electrocatalysis

HER, OER, and overall water splitting electrocatalytic activity were examined for the FeCoNi LTH/MnMoO_4_/GO/FTO composite, as well as for FeCoNi LTH/FTO, MnMoO_4_/FTO, and GO/FTO components individually. The polarization curves are adjusted to account for the ohmic potential drop, and the potentials presented are relative to the RHE. The efficiency of electrocatalytic water splitting is fundamentally constrained by several pivotal parameters, such as a low overpotential, minimal Tafel slope, high *C*_dl_, and reduced *R*_ct_, which are critical metrics for assessing performance, summarized in Table 1, as will be discussed hereafter.

**Table 1:** OER and HER Electrocatalytic Data for the Nanocatalysts

	OER				HER			
catalysts	η_10_ (mV)	η_20_ (mV)	Tafel (mV dec^–1^)	*R*_ct_ (kΩ)	η_10_ (mV)	η_20_ (mV)	**Tafel** **(mV dec** ^–1^ **)**	*R*_ct_ (kΩ)
FeCoNi LTH/MnMoO_4_/GO/FTO	238	260	53	44.8	92	151	46	15.3
FeCoNi LTH/MnMoO_4_/FTO	272	300	65	57.1	167	205	62	40.4
FeCoNi LTH/FTO	310	340	89	76.6	218	263	78	58.2
MnMoO_4_/FTO	340	430	91	142.8	353	–	104	93.9
GO/FTO	420	580	175	155.7	456	–	157	136.8
FTO	690	–	248	253.6	666	–	230	208.7

First, the performance of the synthesized catalysts was evaluated toward the OER by LSV conducted at a scan rate of 5 mV s^–1^ in a 1 mol L^–1^ KOH solution at pH 14, as illustrated in Figure 7a. The FeCoNi LTH/FTO electrocatalyst exhibits a distinctive peak within the potential range of 1.2 to 1.4 V attributed to the oxidation of nickel from lower oxidation states (Ni^0^ or Ni^2+^) to higher oxidation states (Ni^3+^ or Ni^4+^).^59^ A peak around 1.28 V was observed for MnMoO_4_/FTO, probably attributed to Mn^2+^ to Mn^3+^ or from Mn^3+^ to Mn^4+^ redox processes. In contrast, these Ni and Mn oxidation peaks are absent in the FeCoNi LTH/MnMoO_4_/FTO and FeCoNi LTH/MnMoO_4_/GO/FTO electrocatalysts (zoom shown in Figure S2a), likely due to the significant influence of GO that alters the electrochemical environment or to dilution effect. To mitigate the impact of the oxidation peak on the overpotential measurements at lower voltages, the reverse LSV method, scanning from lower to higher voltages, was employed as recommended in the literature.^60^

**Figure 7:**
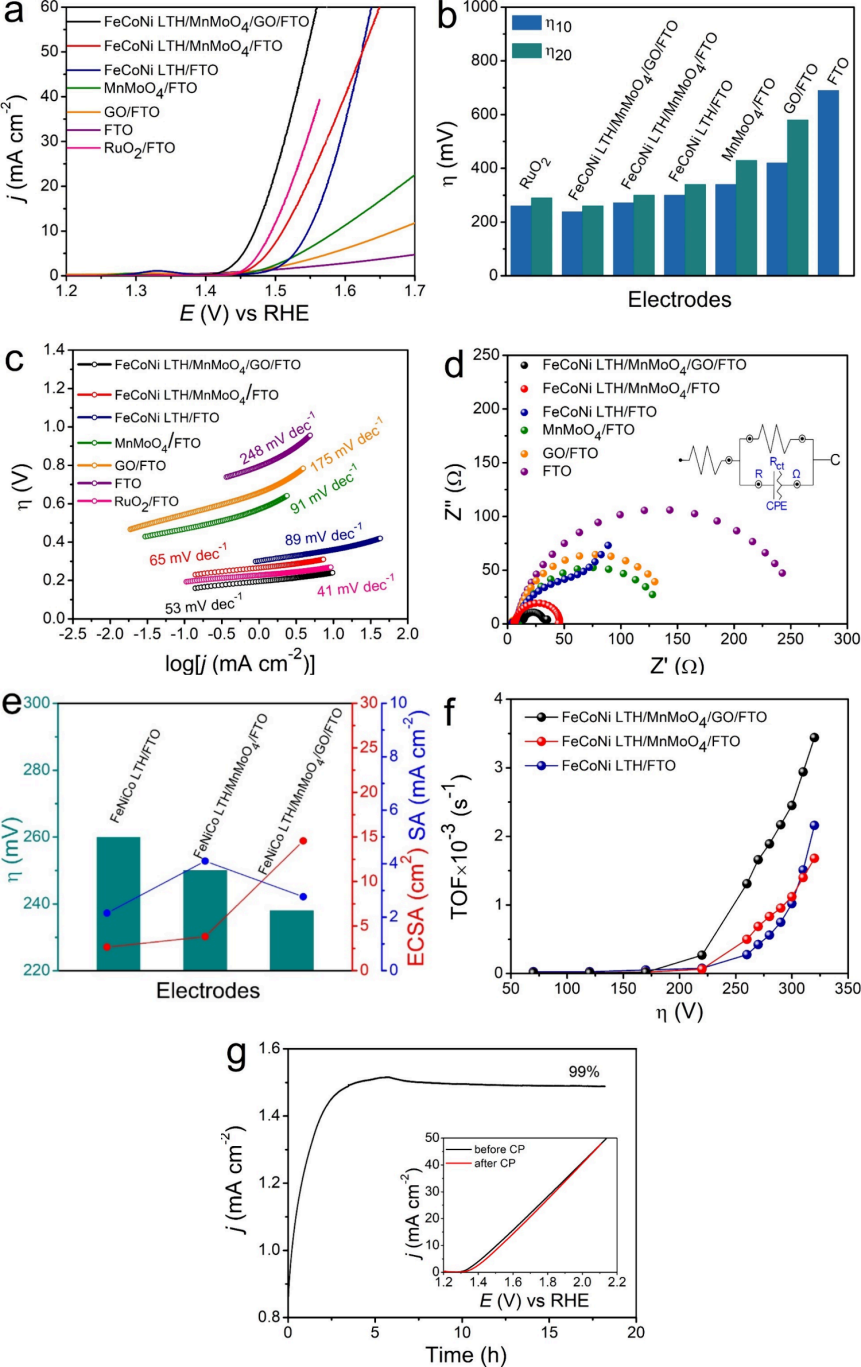
Electrochemical measurements for the OER of the nanocatalysts. (a) LSV curves; (b) histogram of η_10_ and η_20_; (c) Tafel slope plots; (d) EIS Nyquist plots; (e) correlation between η_10_, ECSA, and SA at η = 200 mV; (f) TOF values as a function of η; and (g) chronopotentiometry at 10 mA cm^–2^ (inset: LSV curves before and after chronopotentiometry).

The CV curves illustrate that the Ni^2+^/Ni^3+^ redox couple, typically found within the potential range of 1.35–1.55 V vs RHE, progressively broadens and diminishes in the hybrid electrodes, particularly in the case of FeCoNi LTH/MnMoO_4_/GO/FTO due to a combination with the Mn redox process (Figure S2b). This phenomenon is ascribed to the significant electronic interactions among the Ni, Fe, Co, and Mn species, along with the increased conductivity brought about by GO, which fosters rapid surface reconstruction and facilitates the rapid formation of NiOOH, allowing for its immediate involvement in the OER process instead of a separate redox transition.

The electrocatalyst performance of all electrodes is directly compared in Figure 7b and Table 1 by examining their overpotentials at identical current densities.^61^ The FeCoNi LTH/MnMoO_4_/GO/FTO composite exhibited superior performance in comparison to the individual counterparts, showcasing the benefits of the incorporation of GO and MnMoO_4_ to FeCoNi LTH. The FeCoNi LTH/MnMoO_4_/GO/FTO composite exhibited the lowest overpotentials of 238 and 260 mV at current densities of 10 and 20 mA cm^–2^, respectively. In comparison, FeCoNi LTH/FTO and FeCoNi LTH/MnMoO_4_/FTO achieved higher but still very low overpotential values of 310 and 272 mV at 10 mA cm^–2^ and 340 and 300 mV at 20 mA cm^–2^, respectively. The MnMoO_4_/FTO electrode achieved overpotentials of about 340 and 430 mV at 10 and 20 mA cm^–2^, respectively. At the same current densities, the GO/FTO electrode showed overpotentials of approximately 420 and 580 mV. The bare FTO substrate demonstrated poor OER activity with overpotentials surpassing 690 mV at 10 mA cm^–2^, indicating its limited catalytic effectiveness in the absence of active materials. In comparison, the benchmark RuO_2_ electrode exhibited overpotentials of around 260 and 290 mV at 10 and 20 mA cm^–2^, respectively. Comparatively, the FeCoNi LTH/MnMoO_4_/GO composite attains a slightly improved OER activity in relation to RuO_2_.

The enhanced catalytic efficiency of FeCoNi LTH/MnMoO_4_/GO/FTO can be attributed to the composite’s hierarchical structure that provides an increased surface area and a greater number of accessible active sites, to its rough micronano heterojunction architecture, to the superhydrophilic and underwater superaerophobic properties that facilitate more efficient electron and ion transport as well as aiding in bubble generation and release, and to the synergistic interplay among its constituent elements. Both MnMoO_4_ and FeCoNi-LTH serve as active catalytic centers, as substantiated by previous studies,^27^ while the incorporation of GO not only increases the surface roughness of the composite but also promotes the formation of a heterojunction between FeCoNi LTH and MnMoO_4_, which further augments intrinsic catalytic activity.^31^ A detailed analysis of the overpotentials at current densities of 10 and 20 mA cm^–2^ highlights that the incorporation of GO exerts a slightly more pronounced influence compared to MnMoO_4_. Even a minimal addition of GO significantly enhances the electrode’s conductivity while simultaneously preventing the aggregation of particles, thereby optimizing the exposure of the active sites. Additionally, the synergistic interaction between the manganese and molybdenum centers facilitates electrolyte access and promotes the efficient release of in situ-generated gas bubbles. Furthermore, the presence of multiple oxidation states favors efficient electron transfer and contributes to the stabilization of high-valence catalytic intermediates, which are crucial for optimizing the OER.

To assess the efficiency of the electron transfer of the OER at the electrode interface, the kinetic behavior was analyzed using the Tafel slope, as shown in Figure 7c. A reduced Tafel slope indicates improved OER kinetics, reflecting a more efficient electron transfer process and robust synergistic interactions within the catalyst. The Tafel slopes for FeCoNi LTH/FTO, FeCoNi LTH/MnMoO_4_/FTO, and FeCoNi LTH/MnMoO_4_/GO/FTO electrocatalysts were measured as 89, 65, and 53 mV dec^–1^, respectively. According to the literature, the Tafel slopes for bi- and trimetallic catalysts typically range from 50 to 90 mV dec^–1^, which suggest that the chemical adsorption of hydroxide ions (OH^–^) may be the rate-limiting step in the electrochemical processes occurring at these electrodes.^62,63^ The RuO_2_ electrode displays a lower Tafel slope (∼41 mV dec^–1^), indicative of faster intrinsic kinetics, while the slightly lower overpotential of FeCoNi LTH/MnMoO_4_/GO/FTO is attributed to its higher density of accessible active sites and optimized interfacial structure. The MnMoO_4_/FTO electrode showed a lower Tafel slope of 91 mV dec^–1^ compared to the GO/FTO electrode (175 mV dec^–1^) due to its intrinsic catalytic activity derived from Mn and Mo. Despite having lower conductivity and surface area than GO, MnMoO_4_ offers active metal sites that improve OER kinetics. The incorporation of GO into the FeCoNi LTH/MnMoO_4_/GO/FTO electrocatalyst enhances its surface properties, with GO contributing numerous hydroxyl functional groups. Furthermore, the bare FTO electrode showed a high Tafel slope of about 248 mV dec^–1^, which is suggestive of slow OER kinetics. The lower Tafel slope and reduced overpotential of FeCoNi LTH/MnMoO_4_/GO/FTO compared to the other samples indicate more efficient OER kinetics, which can be attributed to the well-organized, optimized nanocomposite morphology of the electrode, facilitating better electron transfer, strongly coupled effects, and catalytic performance.

A comparative analysis of the overpotentials and Tafel slopes between the synthesized electrodes and similar nonprecious transition metal LTH or MnMoO_4_-based catalysts (Table S4) demonstrated that the FeNiCo LTH/MnMoO_4_/GO/FTO exhibits superior electrocatalytic performance, highlighting its efficiency relative to previously reported systems.^27,40,63,64^ The hierarchical FeCoNi-LTH/NiCo_2_O_4_/CC (CC: carbon cloth) electrode displayed a Tafel slope of 71.5 mV dec^–1^, accompanied by an overpotential of 240 mV at a current density of 50 mA cm^–2^.^64^ The NiCoFe/TX/PW-CNTPE catalyst exhibited a Tafel slope of approximately 83 mV dec^–1^ in alkaline conditions, with an overpotential of 210 mV at 10 mA cm^–2^.^63^ The NiCo_1_Fe_1_ LDH/NF material demonstrated a lower Tafel slope of 59 mV dec^–1^, achieving an overpotential of 231 mV at 10 mA cm^–2^.^40^ Meanwhile, the PANI-MnMoO_4_ catalyst recorded a Tafel slope of around 115.03 mV dec^–1^ in alkaline media, with a higher overpotential of 410 mV at 30 mA cm^–2^.^27^ These findings underscore the competitive electrochemical performance of the catalysts studied herein.

The efficiency of electron transport plays a crucial role in determining the electrochemical catalytic performance, with charge transfer resistance serving as a key indicator of this ability. To explore this aspect, an EIS was employed. The Nyquist plot (Figure 7d) allowed the estimation of the electrolyte resistance (*R*_s_) and the charge transfer resistance (*R*_ct_), which were modeled using an equivalent circuit (Figure 7d-inset). The *R*_s_ and *R*_ct_ values correlate with the kinetics of charge transfer at the solution interface and the electrode/film interface, respectively. Additionally, *C*_dl_ denotes the double layer capacitance associated with electron transfer at the electrode/electrolyte interface, a phenomenon present in all aqueous systems. Notably, *R*_ct_ is indicative of the collective processes governing the OER, directly influencing the overall reaction rate. A reduced semicircle diameter in the Nyquist plot means an accelerated charge transfer rate. The EIS analysis revealed that the *R*_ct_ values for the FeCoNi LTH/FTO, FeCoNi LTH/MnMoO_4_/FTO, and FeCoNi LTH/MnMoO_4_/GO/FTO electrocatalysts were 76.6, 57.1, and 44.8 Ω, respectively. In contrast, MnMoO_4_/FTO, GO/FTO, and bare FTO electrodes showed higher *R*_ct_, i.e., 142.8, 155.7, and 253.6 Ω, respectively. Among these, the FeCoNi LTH/MnMoO_4_/GO/FTO electrocatalyst displayed the smallest semicircle in the Nyquist plot, not only underscoring the synergistic effects of the constituent elements in improving the conductive properties of the multimetal films but also highlighting the role of MnMoO_4_ nanorods and GO in enhancing the electroactive surface area of the FeCoNi LTH electrode. These findings align well with the observed Tafel slope results. This reduction implies enhanced electrical conductivity, an increased charge transfer rate, and improved kinetics for the OER compared to the other catalysts. The observed *R*_ct_ values for the different electrodes can be articulated as follows: FTO > GO/FTO > MnMoO_4_/FTO > FeCoNi LTH/FTO > FeCoNi LTH/MnMoO_4_/FTO > FeCoNi LTH/MnMoO_4_/GO/FTO.

The electrochemically active surface area determines the accessibility to the active sites, which in turn has a direct impact on the efficiency of catalytic reaction.^65^ As presented in Figures S3 and S4, the *C*_dl_ values for FeCoNi LTH/MnMoO_4_/GO, FeCoNi LTH/MnMoO_4_, and FeCoNi LTH were found to be 0.578, 0.153, and 0.10 mF cm^–2^, respectively. The higher *C*_dl_ value of FeCoNi LTH/MnMoO_4_/GO in comparison to the other two electrodes indicates an apparent improvement in the number of active sites. Meanwhile, the ECSA rises in direct proportion to *C*_dl_;^65^ a higher ECSA results in a greater exposure of active sites to the electrolyte and thereby enhances the catalytic activity. In comparison with FeCoNi LTH (2.66 cm^2^) and FeCoNi LTH/MnMoO_4_ (3.82 cm^2^), the FeCoNi LTH/MnMoO_4_/GO composite (14.56 cm^2^) exhibits enhanced catalytic activity as it achieved a higher ECSA (Figure 7e). These findings show that the addition of MnMoO_4_ and GO greatly increases the ECSA, hence improving the efficiency of the catalytic reaction at the electrode.

The specific activity (SA) of the electrodes was evaluated by the normalization of the current at an overpotential of 300 mV by the respective ECSA (Figure 7e) to express the intrinsic activity of the electrochemical accessible active sites of electrodes with different values of ECSA. The SA values are FeCoNi LTH/MnMoO_4_/FTO = 4.1 mA cm^–2^ > FeCoNi LTH/MnMoO_4_/GO/FTO = 2.8 mA cm^–2^ > FeCoNi LTH/FTO = 2.2 mA cm^–2^. Although the composite FeCoNi LTH/MnMoO_4_/GO/FTO presents a higher ECSA than FeCoNi LTH/MnMoO_4_/FTO, it presents a lower SA, which can be explained by the contribution of the GO to increase the roughness and ECSA of the material but does not contribute to the incorporation of active sites. Moreover, the highest SA observed for FeCoNi LTH/MnMoO_4_/FTO confirms the high intrinsic activity of the catalytic sites of FeCoNi LTH and MnMoO_4_ combined due to the synergistic effect between them. Therefore, the lowest overpotential obtained for FeCoNi LTH/MnMoO_4_/GO/FTO is a combination of the high inherent activity of the catalytic sites and also the high ECSA and low charge transfer resistance imputed by the presence of GO. When the current at 300 mV of overpotential is normalized by the loaded catalyst mass, the composite FeCoNi LTH/MnMoO_4_/GO/FTO presents the highest mass activity, indicating a higher number of active sites per gram of catalyst in combination with a higher ECSA. The mass activity of the electrodes followed the order FeCoNi LTH/MnMoO_4_/GO/FTO = 96.2 A g^–1^ > FeCoNi LTH/MnMoO_4_/FTO = 55.2 A g^–1^ > FeCoNi LTH/FTO = 51.5 A g^–1^ (Figure S5). The MA value of the persimmon-like FeOOH-(CrCo)­Ox on the plasma-treated cobalt foam at η = 400 mV was 89.60 A g^–1^, a value inferior to that of FeCoNi LTH/MnMoO_4_/GO/FTO even at a higher overpotential.^9^

The turnover frequency (TOF) was calculated as a function of the overpotential to assess the inherent catalytic activity and kinetics irrespective of the catalyst loading and ECSA.^66^ Additionally, the calculations were based on the catalytic current associated with the O_2_ produced per unit time at a given overpotential and normalized by the amount of metal in the catalyst^67^ for FeCoNi LTH, FeCoNi LTH/MnMoO_4_, and FeCoNi LTH/MnMoO_4_/GO nanocomposites. As shown in Figure 7f, a progressive increase of the TOF with the overpotential indicates the kinetic enhancement of the multielectron OER process. The TOF values of FeCoNi LTH/MnMoO_4_/GO were higher than those of the other catalysts, which significantly emphasize that the composite produces oxygen molecules at a rate more than 2 times higher than that of the other samples, thus confirming the presence of more accessible active sites and higher intrinsic activity. The TOF trend once again demonstrates that our approach to heterostructure formation not only enhances the ECSA but also improves its intrinsic OER performance. These findings highlight the critical role of heterogeneous interface engineering in maximizing the effectiveness of multimetallic active centers during the OER.

The electrochemical stability of FeCoNi LTH/MnMoO_4_/GO/FTO was evaluated by using chronopotentiometry (CP) with a bias of 10 mA cm^–2^ for 18 h. As shown in Figure 7g, there was no significant increase of the overpotential after 18 h, staying at 270 mV, which shows that FeCoNi LTH/MnMoO_4_/GO/FTO nanocomposites can function as highly effective electrocatalysts for the OER with extended operation in alkaline solutions. This improved stability is dependent on the synergistic interaction between the electrocatalyst components that ensure mechanical stability. The minor potential oscillations observed are probably a result of the buildup of oxygen bubbles or localized changes in the electrolyte. Furthermore, the LSV of the OER before and after CP showed no loss of activity (Figure 7g-inset). The XRD and Raman analyses of the sample after chronopotentiometry measurements indicated that the crystalline phase, vibrational, and structural properties of FeCoNi LTH/MnMoO_4_/GO/FTO have been preserved under OER conditions. The diffractogram shows the preservation of the main diffraction peaks relative to the planes (006), (220), (311), (002), (112), (022), and (100) from FeCoNi LTH, MnMoO_4_ and GO (Figure S6). Furthermore, the Raman spectrum (Figure S7) reveals significant changes in the intensity and broadening of the peaks. In particular, the peak intensity of GO decreases. These changes indicate structural rearrangements and transformations of the surface phases during the electrochemical reaction, which are usually accompanied by the formation of amorphous or more active phases, leading to an increased catalytic activity in the OER process.

To further assess the structural stability of the catalyst under the OER conditions, ex situ Raman spectroscopy was conducted both before and after chronoamperometry at a fixed potential of 1.6 V vs RHE for 10 h (Figure S8). The distinct vibrational characteristics of the FeCoNi LTH/MnMoO_4_/GO heterostructure remain largely intact following the operation of the OER, with no new dominant Raman bands that could be indicative of bulk phase transformation. While there are slight variations in intensity, the retention of the characteristic Raman-active modes shows that the overall structural framework maintains its structural integrity during OER.

Moreover, the synthesized electrocatalysts FeCoNi LTH/FTO, FeCoNi LTH/MnMoO_4_/FTO, and FeCoNi LTH/MnMoO_4_/GO/FTO also show promising catalytic activity in HER in 1 mol L^–1^ KOH solution (pH 14), as demonstrated in Figure 8, proving their bifunctional activity toward OER and HER. The LSV curves (Figure 8a) show that FeCoNi LTH/MnMoO_4_/GO/FTO achieves the lowest overpotential of 92 mV at −10 mA cm^–2^ and 151 mV at −20 mA cm^–2^ compared to FeCoNi LTH/MnMoO_4_/FTO (167 and 205 mV) and FeCoNi LTH/FTO (218 and 263 mV), as indicated in the histogram (Figure 8b). The HER performance of FeCoNi LTH/MnMoO_4_/GO/FTO is therefore comparable to that of Pt (73 and 134 mV at −10 and −20 mA cm^–2^), with only a small difference in the overpotential. Such improved performance is attributed to the synergy of the material properties. The hierarchical structure of FeCoNi LTH provides numerous active sites of Fe, Ni, and Co, which are proficient in H* adsorption, to constitute the foundation of the catalytic mechanism. The addition of MnMoO_4_ creates defects and oxygen vacancies, as suggested by its Raman fingerprint, which decrease the energy barrier of the Volmer step via the stabilization of the H* intermediates. The superhydrophilic property and high conductivity of GO also facilitate this effect by promoting electron transport and electrolyte accessibility, while its superaerophobic feature guarantees the effective release of H_2_ bubbles. In contrast, MnMoO_4_/FTO and GO/FTO electrocatalysts demonstrate elevated overpotentials of 353 and 456 mV, respectively, at a current density of −10 mA cm^–2^. This observation highlights their specific constraints, namely, the moderate catalytic activity for HER of MnMoO_4_ and the insufficient number of active sites in GO, whereas the bare FTO exhibits an overpotential of 666 mV, emphasizing its inertness. Compared to the OER data in Figure 7b, the HER overpotentials are significantly lower than those for FeCoNi LTH/MnMoO_4_/GO/FTO at 10 and 20 mA cm^–2^. This difference results from the four-electron OER process, which entails greater energy barriers and the creation of O–O bonds, as opposed to the simpler two-electron HER mechanism. The superior performance toward HER is a confirmation of the maximized electronic structure of the FeCoNi LTH/MnMoO_4_ heterojunction, further augmented by GO and therefore making it particularly effective for hydrogen evolution.

**Figure 8:**
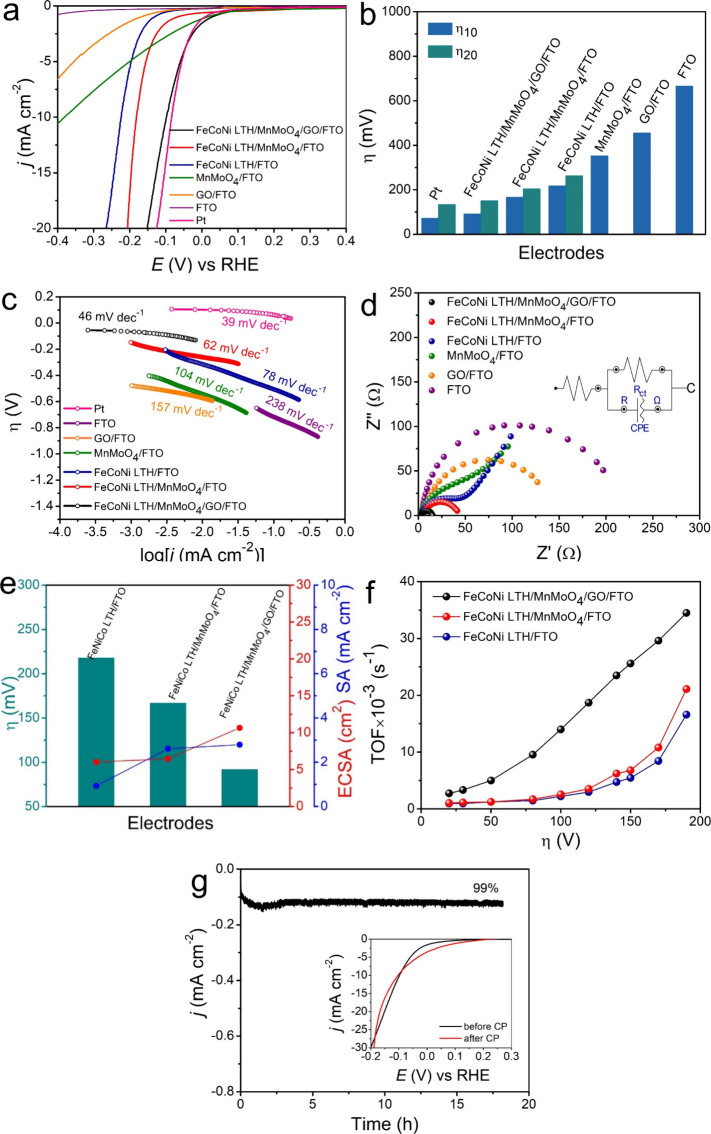
Electrochemical measurements for HER of the nanocatalysts. (a) LSV curves; (b) histogram of η_10_ and η_20_; (c) Tafel slope plots; (d) EIS Nyquist plots; (e) correlation between η_10_, ECSA, and SA at η = 200 mV; (f) TOF values as a function of η; and (g) chonopotentiometry at −10 mA cm^–2^ (inset: LSV curves before and after chronopotentiometry).

The HER Tafel slopes exhibit a progressive decrease from the individual components to the composites (Figure 8c). FTO shows a high value of 230 mV dec^–1^, and upon the introduction of GO, this value drops to 157 mV dec^–1^, indicating a moderate kinetic enhancement through conductivity. The decrease to 104 mV dec^–1^ by MnMoO_4_ is most likely because of the easier charge transfer accomplished by its redox sites. The use of the FeCoNi LTH leads to a further decrease to 78 mV dec^–1^, which aligns with the Volmer–Heyrovsky mechanism. The combination of FeCoNi LTH and MnMoO_4_ results in a Tafel slope of 62 mV dec^–1^, indicating synergistic effects, while the incorporation of GO into the composite FeCoNi LTH/MnMoO_4_/GO/FTO achieves the lowest Tafel slope of 46 mV dec^–1^, highlighting the optimization of the kinetics through enhanced electron mobility provided by GO. For reference, the Pt electrode demonstrates a reduced HER Tafel slope of approximately 39 mV dec^–1^, indicating its enhanced intrinsic kinetics for hydrogen adsorption and desorption; however, the FeCoNi LTH/MnMoO_4_/GO/FTO catalyst nearly reaches this benchmark performance due to improvements in charge transfer and active site induced by its heterostructure.

The HER activity of the FeCoNi LTH/MnMoO_4_/GO/FTO electrocatalyst is compared to those of representative transition-metal-based electrocatalysts shown in Table S5, highlighting the exceptional high activity for platinum group metal (PGM)-free catalysts. For example, the B-doped coral-like hierarchical nanoarray B_10_–FeCoNi-LDH over NF exhibited an overpotential of 56 mV at −10 mA cm^–2^ and Tafel slope of 46 mV dec^–1^,^68^ while NiCoFe LTHs supported on carbon fiber cloth displayed 200 mV and 70 mV dec^–1^.^69^ CoMoO_4_/MnMoO_4_ on NF achieved 153 mV of η_10_ and 86.28 mV dec^–1^ of Tafel slope.^70^

The parallel decrease in *R*_ct_ for both reactions (OER and HER) for the composite in relation to the pure components highlights the significance of GO and MnMoO_4_ in improving the conductivity and interfacial kinetics. Nevertheless, the lower *R*_ct_ observed in FeCoNi LTH/MnMoO_4_/GO/FTO for the HER (15.30 Ω) (Figure 8d) compared to that of the OER (44.81 Ω) suggests a more effective electron transfer process. This difference can be attributed to the reductive environment of the HER, which inhibits oxide formation, thereby maintaining the conductive properties of the catalyst and enhancing the impact of GO. Conversely, the oxidative conditions of the OER facilitates the development of insulating oxide layers, resulting in increased resistance. Comprehensive impedance fitting parameters (*R*_s_, *R*_ct_, CPE, *n*, and χ^2^) derived from the equivalent circuit modeling are presented in Table S6.

The capacitive behavior controlled by *C*_dl_ and ECSA is reflected in the HER current density vs scan rate plot (Figures S9 and S10), which displays a linear increase in current density with scan rate. The *C*_dl_ was measured in a non-Faradaic region of CV curves within a potential range of −0.30 to 0.00 V vs SCE at various scan rates ranging from 5 to 300 mV s^–1^. The FeCoNi LTH/MnMoO_4_/GO/FTO composite exhibits the highest *C*_dl_ value of 0.426 mF cm^–2^, which is significantly greater than the *C*_dl_ values of the other samples, specifically 0.259 mF cm^–2^ for FeCoNi LTH/MnMoO_4_/FTO and 0.242 mF cm^–2^ for FeCoNi LTH/FTO. The corresponding ECSA for FeCoNi LTH/MnMoO_4_/GO/FTO is 10.65 cm^2^, which is significantly higher than that of FeCoNi LTH/MnMoO_4_ (6.46 cm^2^) and FeCoNi LTH (6.04 cm^2^), respectively. The integration of MnMoO_4_ into FeCoNi LTH results in an increased *C*_dl_ and ECSA due to a rougher surface and more redox-active sites. The defect-rich FeCoNi LTH/MnMoO_4_/GO composite shows a notable increase in active site density, which highlights the beneficial effect of GO to improve electrochemical performance for HER. The SA values obtained for the materials at an overpotential of 200 mV (Figure 8e) are FeCoNi LTH/MnMoO_4_/GO/FTO = 2.79 mA cm^–2^ > FeCoNi LTH/MnMoO_4_/FTO = 2.61 mA cm^–2^ > FeCoNi LTH/FTO = 0.93 mA cm^–2^; the higher values obtained show the enhanced intrinsic activity of the electrodes when both catalytic sites of FeCoNi LTH and MnMoO_4_ are present in combination with GO. The mass activity of the catalysts for HER at η = 200 mV are FeCoNi LTH/MnMoO_4_/GO/FTO = 47.2 A g^–1^ > FeCoNi LTH/MnMoO_4_/FTO = 36.6 A g^–1^ > FeCoNi LTH/FTO = 20.1 A g^–1^ (Figure S11). The higher MA of FeCoNi LTH/MnMoO_4_/GO/FTO is a reflection of more HER active sites per gram of catalyst and a higher ECSA, favoring the overall activity exhibited by the composite. Yttrium- and nitrogen-doped NiCo phosphide nanosheets N-YNiCoP/PNCF showed MA at η = 200 mV of 22.65 A g^–1^,^8^ lower than FeCoNi LTH/MnMoO_4_/GO/FTO.

As illustrated in Figure 8f, all catalysts demonstrate comparable TOF values at low overpotentials, suggesting comparable intrinsic HER activity per active site, which aligns with a Volmer-dominated reaction regime. At higher cathodic overpotentials, the TOF experiences a notable increase, especially for the FeCoNi LTH/MnMoO_4_/GO/FTO. Significantly, the potential-dependent TOF trends aligns well with both the HER activity derived from LSV and the ICP-OES results, reaffirming that the improved HER performance of FeCoNi LTH/MnMoO_4_/GO/FTO originates from stabilized active sites and accelerated reaction kinetics rather than catalyst degradation.

The stability of the FeCoNi LTH/MnMoO_4_/GO/FTO catalyst under HER conditions was evaluated by chronopotentiometry for 18 h at −10 mA cm^–2^ (Figure 8g). The high current density was sustained at a potential of about 100 mV with few fluctuations, showcasing the exceptional stability of the FeCoNi LTH/MnMoO_4_/GO/FTO electrocatalyst for HER catalysis in alkaline solutions. HER LSV before and after CP shows no loss of activity (inset of Figure 8g). The XRD analysis of the FeCoNi LTH/MnMoO_4_/GO/FTO electrocatalyst after the long-term HER test indicated that their crystalline phase and diffraction peaks had been preserved and confirmed the initially existing significant diffraction peaks for the (003), (006), (021), (220), (311), (002), (400), (022), and (100) planes from FeCoNi LTH, MnMoO_4_, and GO (Figure S12). Figure S13 displays the Raman spectrum following HER, showing a reduction in peak intensity and broadened signals. These alterations suggest that structural rearrangements and surface phase transformations have occurred due to the HER, which are typically linked to the formation of amorphous or more catalytically active phases, ultimately improving the HER performance.

Overall, the improved performance of the HER and the OER in the FeCoNi LTH/MnMoO_4_/GO/FTO hybrid electrode can be ascribed to the synergistic interaction among its constituent components. The FeCoNi LTH serves as the main catalytic framework, providing numerous redox-active metal centers where Ni and Co undergo reversible oxidation to form catalytically active NiOOH and CoOOH species for the OER, while Fe^3+^ modulates the electronic structure and facilitates oxygen intermediate formation. MnMoO_4_ contributes with additional Mn^3+^/Mn^4+^ redox sites and defect-induced oxygen vacancies, which enhance OH^–^ adsorption and stabilize intermediates during both HER and the OER, thus speeding up reaction kinetics. At the same time, GO functions as an effective electron-conducting matrix, enhancing interfacial charge transfer, ensuring the efficient use of active sites, and improving electrolyte accessibility. The robust interfacial interaction among FeCoNi LTH, MnMoO_4_, and GO alters the local electronic configuration, decreases the energy barrier for rate-determining steps, and accelerates reaction kinetics, as evidenced by the decreased Tafel slopes and charge transfer resistance. This synergistic effect yields enhanced catalytic performance in comparison to the separate components, illustrating a true synergistic catalytic mechanism.

Considering all of that, the FeCoNi LTH/MnMoO_4_/GO/FTO electrode exhibited promising performance in both the OER and HER, showcasing its significant potential for application as a bifunctional electrode in water decomposition devices. Figure 9a presents the LSV curves of the overall water splitting using FeCoNi LTH/MnMoO_4_/GO/FTO in both electrodes of a two-electrode cell, which exhibited exceptional activity. Furthermore, RuO_2_/FTO electrodes with equivalent loadings were also evaluated for comparative analysis. Remarkably, the FeCoNi LTH/MnMoO_4_/GO/FTO electrode only requires a voltage of 1.57 V to achieve 10 mA cm^–2^ current density in a water splitting system, which is much lower than the 1.65 V of RuO_2_/FTO-based overall water electrolysis, therefore showing that FeCoNi LTH/MnMoO_4_/GO/FTO achieved an outstanding overall hydrolysis performance. A comparison with other bifunctional electrocatalysts is presented in Table S7. It is notable that the FeCoNi LTH/MnMoO_4_/GO/FTO-based overall water splitting exhibits a lower or comparable cell voltage than those of other bifunctional electrocatalysts; for instance, NiCoFe LTHs supported on carbon fiber cloth exhibited a cell voltage of 1.55 V,^68^ while ZIF-67-derived FeCoNi LDH with a 3D nanoflower hierarchical structure achieved 1.70 V.^69^ Finally, to comprehend the stability of the FeCoNi LTH/MnMoO_4_/GO/FTO electrocatalyst for overall water splitting, a long-term electrochemical stability test was performed by chronoamperometry in a 1 mol L^–1^ KOH electrolyte at 1.57 V for 31 h, in which the current density exhibited minimal attenuation, retaining 88% of its initial value as shown in Figure 9b. The high stability is due to the electrodeposition of the solid catalysts to the FTO electrode instead of other common methods of deposition such as drop casting, spin coating, and dipping coating, among others. The electrodeposition allows for a high adhesion of the catalysts to the FTO substrate.

**Figure 9:**
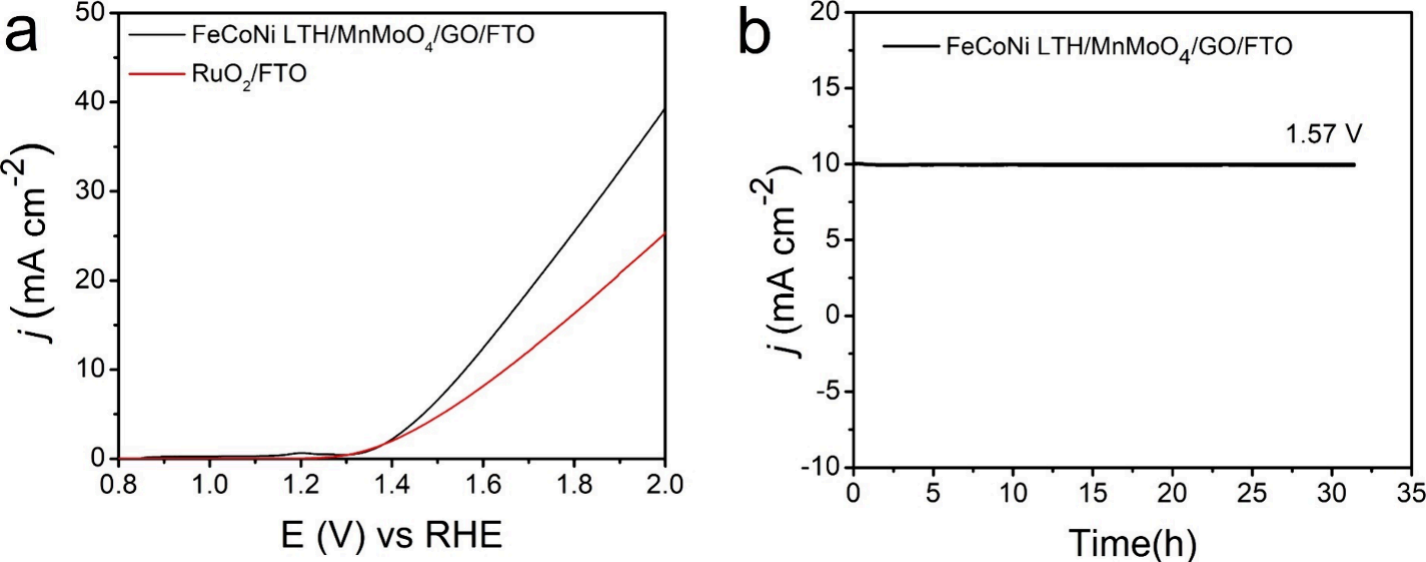
(a) Polarization curves of the overall water splitting using FeCoNi LTH/MnMoO_4_/GO/FTO and RuO_2_/FTO as functional electrocatalysts (same loading) in a two-electrode system at the scan rate of 5.0 mV s^–1^ and (b) *i*–*t* curve of FeCoNi LTH/MnMoO_4_/GO/FTO at 1.57 V for 31 h.

ICP-OES analyses were carried out to quantitatively evaluate the elemental composition of the catalysts before (Tables S8 and S9) and after overall water splitting (Tables S10 and S11) extended electrochemical stability testing, offering direct insight into the behavior of metal retention and dissolution under alkaline operating conditions. The ICP-OES analysis conducted prior to the stability test on the pristine FeCoNi LTH electrode shows the successful integration of all three transition metals, aligning with the designed trimetallic hydroxide framework. It is possible to notice that the Fe/Co/Ni molar ratio stayed at around 1:0.64:0.67 in the FeCoNi LTH and also in the composites, with small variations. In relation to the Mn/Mo molar ratio of around 1:0.70, a negligible variation was also observed. Following prolonged electrochemical operation, dissolution of Fe, Co, and Ni was detected, indicating partial metal leaching from the LTH lattice. This phenomenon is typically associated with the inherent structural flexibility of layered hydroxides in alkaline environments, where surface reconstruction and metal dissolution may occur during extended OER/HER cycling. Importantly, the degree of metal loss is relatively moderate, indicating that the LTH framework retains a significant portion of its active metal centers even after extended operation. Upon the introduction of MnMoO_4_, a significant improvement in metal retention is noted following stability assessments in the FeCoNi LTH/MnMoO_4_ heterostructure. ICP-OES analysis indicates a reduced dissolution of Fe, Co, and Ni in comparison to that of the unmodified LTH, along with small leaching of Mn and Mo. This improved stability is likely due to the establishment of a heterointerface between LTH and MnMoO_4_, which facilitates stronger electronic interactions and structural confinement of the active metals. Furthermore, the MnMoO_4_ phase plays a critical stabilizing role by forming strong interfacial metal–oxygen–metal linkages (M–O–M), which effectively anchor the hydroxide domains and mitigate metal detachment under alkaline conditions. In addition, the multivalent nature of Mn and Mo is anticipated to play a role in charge compensation throughout the electrochemical operation, thus reducing the excessive metal dissolution from the LTH phase. The FeCoNi LTH/MnMoO_4_/GO/FTO electrode exhibits the highest compositional stability among all investigated catalysts. The ICP-OES analysis conducted following stability testing reveals that there is minimal dissolution of all constituent metals, underscoring the essential function of graphene oxide in stabilizing the heterostructure. The addition of GO results in a conductive and mechanically strong matrix that improves interfacial adhesion, prevents catalyst detachment, and promotes uniform charge distribution across the surface of the electrode. Moreover, the synergistic interaction among FeCoNi LTH, MnMoO_4_, and GO effectively limits local overoxidation and metal dissolution during long-term operation. Furthermore, the synergistic interaction between LTH, MnMoO_4_, and GO promotes interfacial bonding and decreases stress induced by local overpotential, thus reducing structural degradation during prolonged operation. A comparative assessment of the three systems distinctly illustrates a gradual improvement in both structural and chemical stability following the order FeCoNi LTH < FeCoNi LTH/MnMoO_4_ < FeCoNi LTH/MnMoO_4_/GO (Table S11). This trend is closely associated with the electrochemical stability and activity retention noted during extended testing of the HER and the OER, thereby affirming that interfacial engineering and carbon integration are crucial in mitigating metal leaching while maintaining catalytic performance.

Figure S14 shows the SEM image of the FeCoNi LTH/MnMoO_4_/GO nanohybrid following the stability test at 1.57 V for 31 h. In comparison to that of the pristine catalyst (Figure 2), the overall hierarchical morphology is largely maintained. The spherical FeCoNi LTH nanoparticles and MnMoO_4_ nanorods preserve their unique shapes and sizes with no notable fragmentation or morphological deformation detected. A slight condensation or restacking of the GO layers is apparent, likely resulting from the extended exposure to alkaline electrolyte and gas evolution forces. Nevertheless, the nanoparticles and nanorods remain securely anchored to the GO surface with minimal agglomeration, thereby confirming the strong structural integrity of the heterostructure and its remarkable long-term stability under severe operating conditions. As illustrated in Figure S15, the Raman spectrum after the stability test demonstrates the continued presence and even enhancement of distinct vibrational peaks associated with FeCoNi LTH and MnMoO_4_. Simultaneously, minor shifts or variations in intensity are noted in the D and G bands of GO, suggesting a moderate structural condensation of the graphene layers. These observations indicate the remarkable structural integrity of both nanoparticle phases and imply the in situ generation of highly active surface species during the stability test, thereby reinforcing the durability of the composite under operational conditions.

Finally, the stability of the FeCoNi LTH/FTO and FeCoNi LTH/MnMoO_4_/FTO electrocatalysts for overall water splitting was also tested at 1.57 V for 31 h, and the current density exhibited minimal attenuation, as shown in Figure S16, showcasing the high stability of the nanocatalysts.

## Conclusions

This study introduces a significant advancement in the creation of bifunctional electrocatalysts aimed at achieving highly efficient overall water splitting through the design and synthesis of a hierarchical FeCoNi LTH/MnMoO_4_/GO nanohybrid electrodeposited onto FTO substrates. The existence of different metal atoms in various valence states within the catalyst creates a synergistic interplay among FeCoNi LTH nanoparticles, MnMoO_4_ nanorods, and GO nanosheets, with a prevalence of Ni^2+^, Co^3+^, Fe^2+^, Mn^4+^, and Mo^6+^ at the surface, resulting in a defect-rich heterostructure that greatly improves electrocatalytic performance by optimizing active site density, electrical conductivity, and charge transfer kinetics. The heterostructure configuration plays an important role in creating numerous interfaces and favorable synergistic effects for effective water electrolysis. The resulting FeCoNi LTH/MnMoO_4_/GO/FTO electrocatalyst shows excellent performance, achieving low overpotentials of 238 mV for OER and 92 mV for HER at 10 mA cm^–2^ in 1 mol L^–1^ KOH, with optimal Tafel slopes of 53 and 46 mV dec^–1^, respectively. These metrics, combined with excellent stability over 31 h of continuous electrolysis with minimal attenuation and exceptional bifunctional catalytic performance, establish the electrode as a superior alternative compared to noble-metal-based catalysts like RuO_2_, necessitating only 1.57 V to drive overall water splitting at 10 mA cm^–2^. The incorporation of GO into the FeCoNi LTH/MnMoO_4_ nanohybrid not only addresses the intrinsic conductivity challenges of both LTH and MnMoO_4_ but also significantly improves the electrochemically active surface area. The defect-rich heterostructure, along with the synergistic interactions among its Earth-abundant constituents, provides remarkable electrocatalytic performance, cost-effectiveness, and strong chemical stability, effectively tackling the challenges of slow kinetics and durability in alkaline water electrolysis. Through the application of scalable synthesis techniques and innovative design approaches, including self-supported structures to avoid using a binder, multimaterial synergies, and structure assembly for superior mass transfer, this study establishes a transformative framework for the development of low-cost, high-performance bifunctional electrocatalysts, thereby paving the way for efficient hydrogen production and the wider adoption of green energy technologies.

## Supplementary Material



## Notes

Additional characterization data (including EDS, XPS, XRD, Raman, and SEM images before or after stability tests), long-term stability tests, ICP-OES analysis, *C*_dl_ and ECSA measurements, and electrocatalytic parameters from OER, HER, and overall water splitting from related materials described in the literature (PDF)
