# Supplementary material for: The Hierarchical Trimetallic FeCoNi LTH/MnMoO4/GO Nanohybrid as an Active Catalyst with Enhanced Bifunctional Performance for Efficient Overall Water Splitting
Source: ACS Appl Mater Interfaces. 2026 Mar 7;18(10):14914–32. doi: 10.1021/acsami.5c20919 (PMC13006961; doi:10.1021/acsami.5c20919)
Supplement: Supplementary file 1 [file am5c20919_si_001.pdf]

Supporting information for

The Hierarchical trimetallic FeCoNi LTH/$MnMoO_4$/GO Nanohybrid as an active catalyst with Enhanced Bifunctional Performance for Efficient Overall Water Splitting

Fahimeh Sadat Vajedi[a*], Yutao Xing[b], Nakédia M. F. Carvalho[a*]

[a] *Universidade do Estado do Rio de Janeiro, Instituto de Química, Rua São Francisco Xavier, 524, Maracanã, Rio de Janeiro, RJ, 20550-013, Brazil*

[b] *Universidade Federal Fluminense, Instituto de Física, Ave. Gal. Milton Tavares de Souza, Campus Praia Vermelha, São Domingos, Nitéroi 24210-346, Brazil*

*Email: nakedia@uerj.com

*Email: vajedi.fahimeh@ce.uerj.br

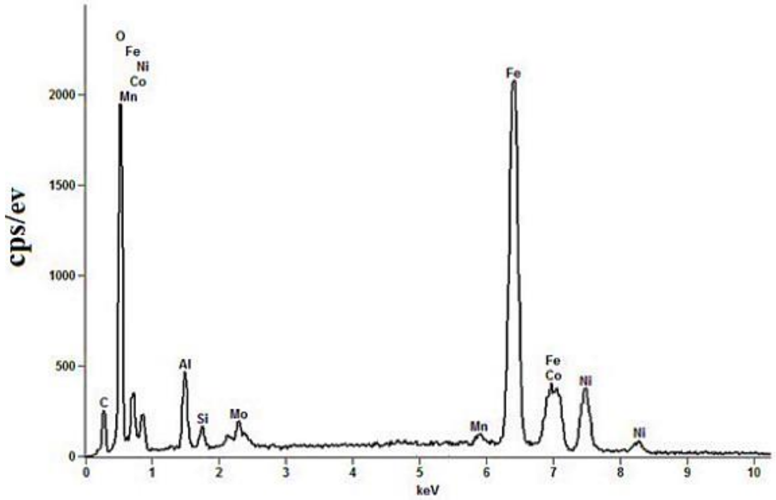

Fig. S1. EDS spectra of FeCoNi LTH/$MnMoO_4$/GO.

Table S1. XPS peak fitting parameters for the FeCoNi LTH/$MnMoO_4$/GO nanohybrid.

Elements	Attribution	FWHM (eV)	Area	Positions(eV)	Atom (%)
C1s	C=C	1.43	15930.88	284.8	47.08
	C-C	1.40	8487.50	285.9	26.48
	C-O	1.71	5354.99	286.75	16.56
	C=O	2.14	3035.70	288.9	9.13
O1s	M-O	1.33	16727.98	529.6	10.13
	C=O	1.89	73830.03	530.6	44.71
	C-OH	1.54	38695.14	531.9	23.44
	C-O	2.46	35859.46	533	21.73
Ni2p3/2	Ni^{2+}	2.18	25717.65	855.67	44.86
	Ni^{3+}	2.36	6313.34	857.75	11.02
	satellite	3.02	8617.11	861.40	15.08
Ni2p1/2	Ni^{2+}	1.44	4915.42	873.40	8.66
	Ni^{3+}	1.90	2643.01	874.40	4.66
	satellite	4.31	8898.39	879.8	15.73
Fe2p3/2	Fe^{0}	3.01	2488.54	705.7	2.20
	Fe^{2+}	4.44	32581.59	711.13	28.86
	Fe^{3+}	6.53	30791.64	712.79	27.29
	satellite	6.68	13769.95	716.16	12.22
Fe2p1/2	Fe^{0}	9.30	13764.47	719.28	12.23
	Fe^{2+}	3.87	10121.54	723.87	9.01
	Fe^{3+}	4.38	6585.93	726.24	5.78
	satellite	6.49	2617.23	732.90	2.34
Co2p3/2	Co^{0}	5.62	4218.32	775.35	8.78
	Co^{2+}	2.7	14804.61	780.84	30.90
	Co^{3+}	4.92	9865.93	784.64	20.62
	satellite	5.91	8090.50	786.4	16.93
Co2p1/2	Co^{0}	2.15	2234.90	795.47	4.69
	Co^{2+}	1.78	2436.77	796.84	5.12
	Co^{3+}	6.54	3338.71	798.87	7.02
	satellite	4.85	2812.50	804.08	5.93
Mn2p3/2	Mn^{3+}	2.93	60014.08	642.25	39.63
	Mn^{4+}	6.27	48984.80	643.40	32.36
Mn2p1/2	Mn^{3+}	4.09	33310.39	653.71	22.07
	Mn^{4+}	2.56	8955.16	655.37	5.94
Mo $3d_{5/2}$	Mo^{6+}	1.2	1186.80	232.33	64.39
Mo $3d_{3/2}$	Mo^{6+}	1	656.05	235.44	35.61

Table S2. XPS atomic percentage for the survey spectrum for FeCoNi LTH/$MnMoO_4$/GO.

Element	FWHM (eV)	Area	Positions (eV)	Atoms (%)
C1s	2.15	102705.01	285.08	32.40
O1s	3.77	462315.80	530.08	47.58
Ni2p	3.18	164730.09	856.08	2.34
Fe2p	4.92	171697.19	711.08	3.30
Co2p	5.45	212114.18	781.08	3.49
Mn2p	3.82	383207.89	642.08	8.59
Mo3d	1.2	14091.4	232.83	2.3

Table S3. XPS data for FeCoNi LTH/$MnMoO_4$/GO.

Elements	Attribution	FWHM (eV)	Area	Positions (eV)	Atoms (%)	Oxidation-state ratios
$Ni2p_{3/2}$	Ni^{2+}	2.18	19436.21	855.67	51.96	63.76
	Ni^{3+}	2.36	11043.53	857.75	29.54	36.23
	Ni^{2+}/Ni^{3+}	-	-	861.40	-	1.76
$Fe2p_{3/2}$	Fe^{2+}	4.44	14823.78	711.13	56.09	59.53
	Fe^{3+}	6.53	10075.17	712.79	38.15	40.46
	Fe^{2+}/Fe^{3+}	-	-	-	-	1.47
$Co2p_{3/2}$	Co^{2+}	2.7	8527.77	780.84	30.90	41.69
	Co^{3+}	4.92	11925.10	784.64	42.33	58.30
	Co^{2+}/Co^{3+}	-	-	-	-	0.72
$Mn2p_{3/2}$	Mn^{3+}	2.93	27669.32	642.25	36.63	36.1
	Mn^{4+}	4.27	48984.80	643.40	63.82	64
	Mn^{3+}/Mn^{4+}	-	-	-	-	0.57
Mo $3d_{5/2}$	Mo^{6+}	1.2	1425.15	232.33	53.38	
Mo $3d_{3/2}$	Mo^{6+}	186.	1244.07	235.44	46.62	

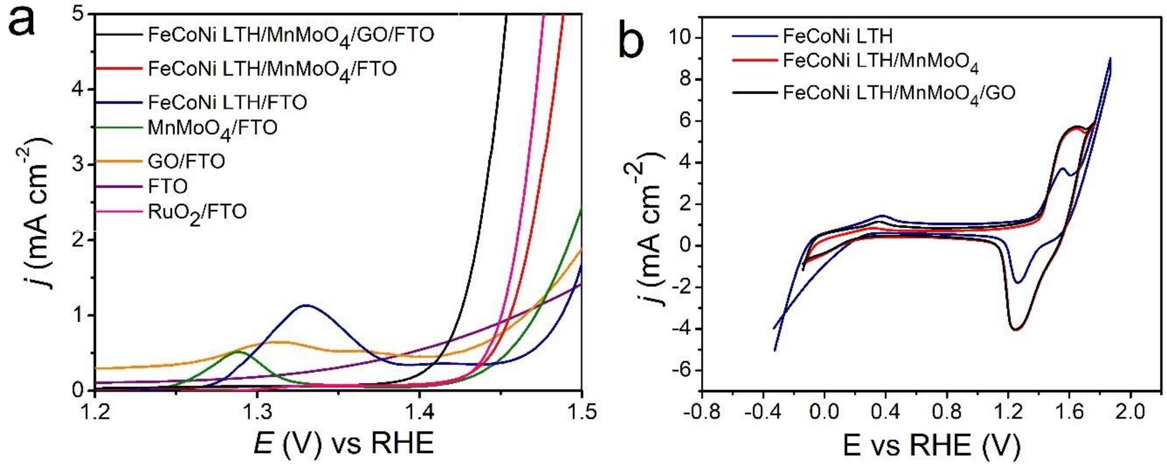

Fig. S2. a) Zoom of Fig. 7a. b) FeCoNi LTH/$MnMoO_4$/GO/FTO electrodes recorded in 1.0 mol L^{-1} KOH at a scan rate of 5 mV s^{-1}.

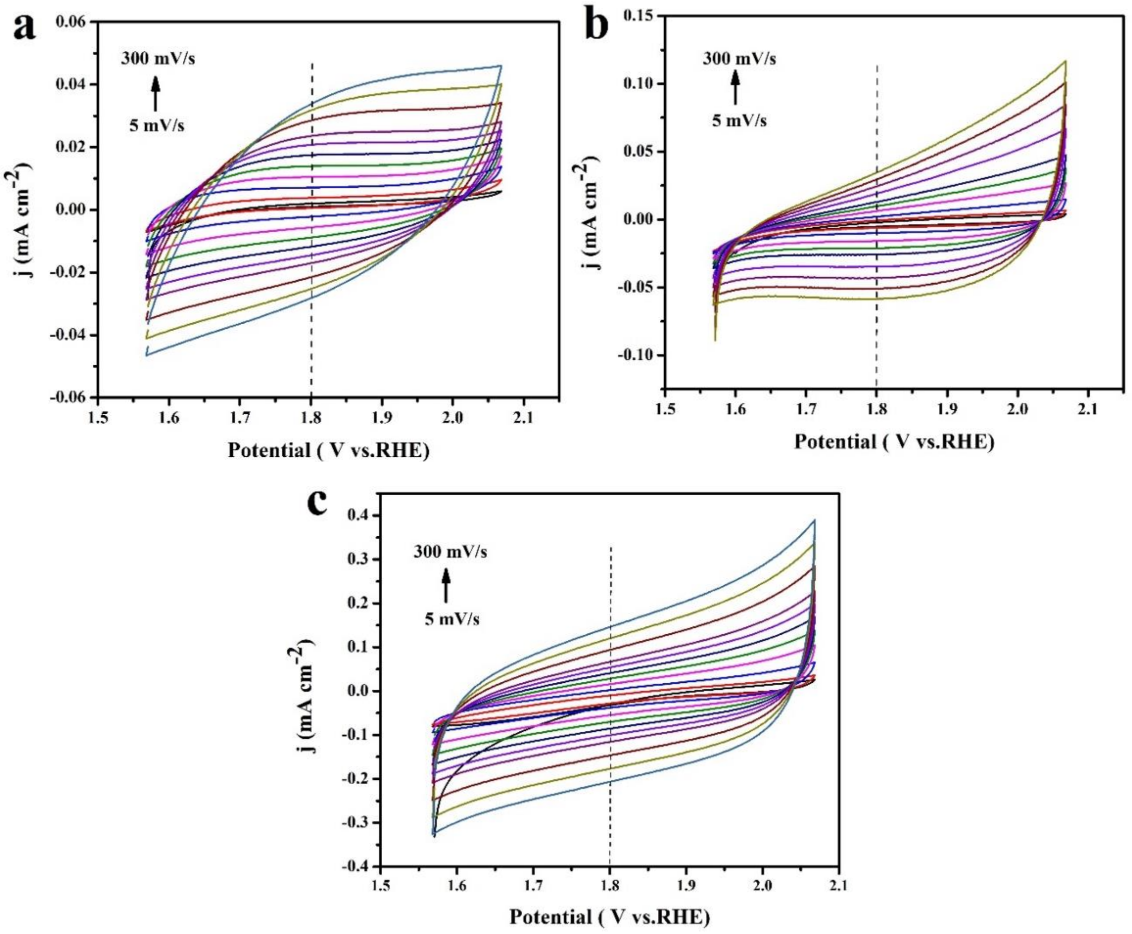

Fig. S3. CV curves of (a) FeCoNi LTH/FTO, (b) FeCoNi LTH/$MnMoO_4$/FTO, and (c) FeCoNi LTH/$MnMoO_4$/GO/FTO measured in 1.0 mol L^{-1} KOH in the non-Faradaic potential window for OER.

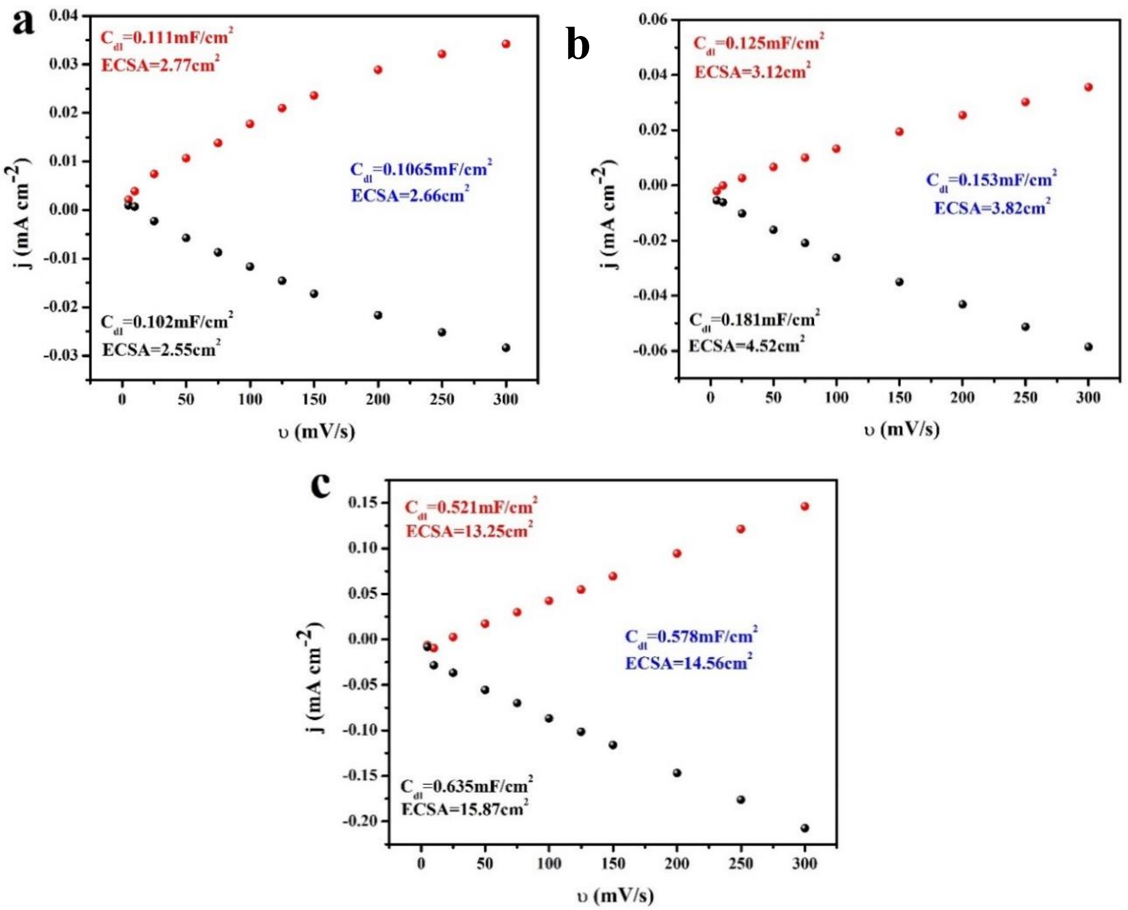

Fig. S4. Corresponding plots of the current density versus the scan rate for (a) FeCoNi LTH/FTO, (b) FeCoNi LTH/$MnMoO_4$/FTO, and (c)FeCoNi LTH/$MnMoO_4$/GO/FTO for OER.

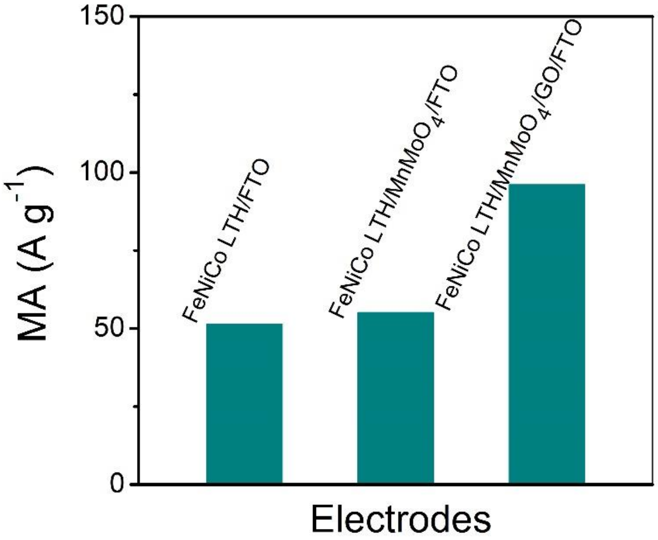

Fig. S5. Mass activity values at η = 300 mV for OER.

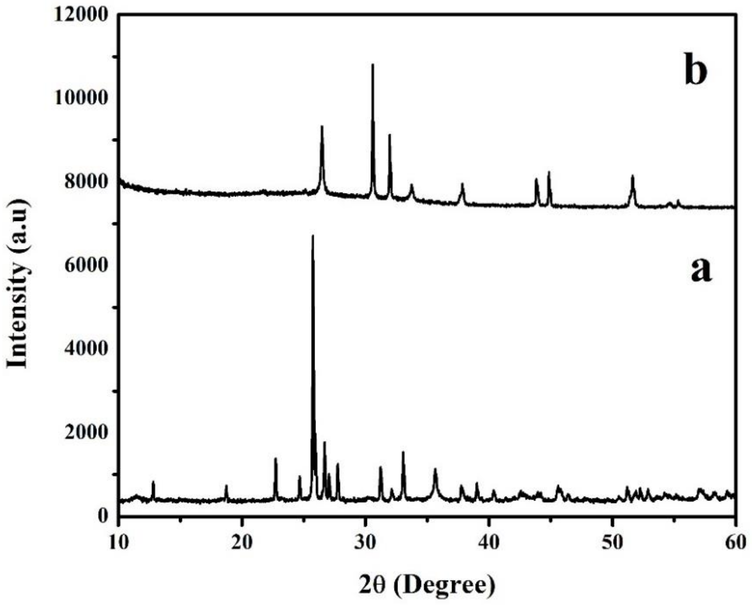

Fig. S6. XRD pattern for FeCoNi LTH/$MnMoO_4$/GO/FTO before (a) and after (b) stability in OER tested by chronopotentiometry at 10 mA cm^{-2} for 18 h referred to Fig. 7g.

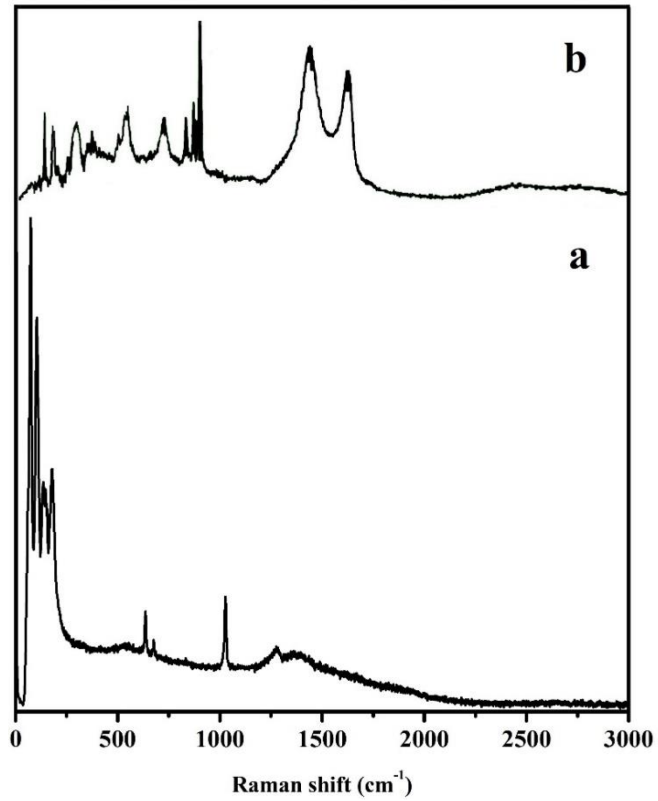

Fig. S7. Raman spectroscopy of FeCoNi LTH/$MnMoO_4$/GO/FTO after (a) and before (b) 18 h stability in OER tested by chronopotentiometry at 10 mA cm^{-2} for 18 h referred to Fig. 7g.

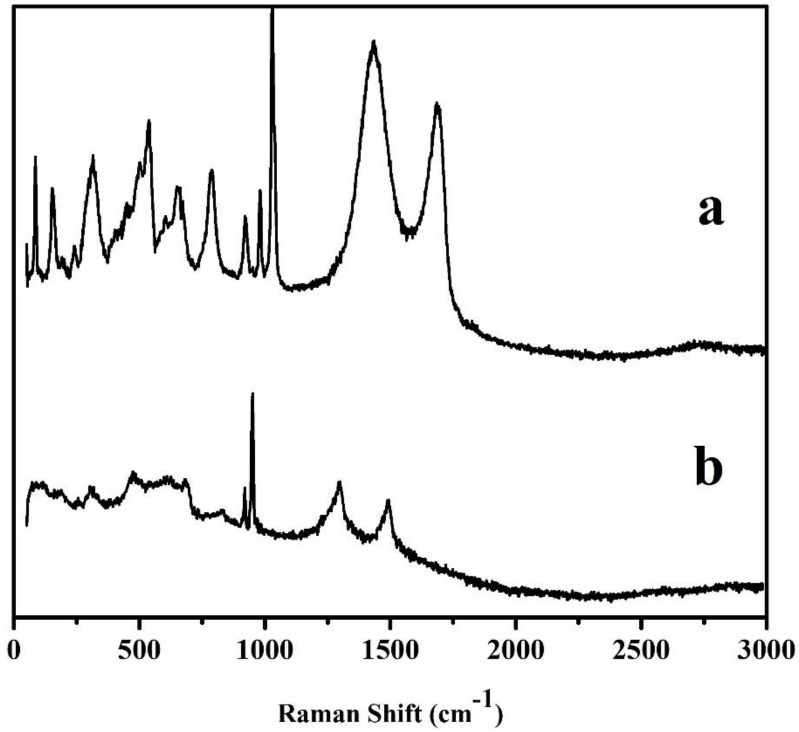

Fig. S8. Raman spectroscopy of FeCoNi LTH/$MnMoO_4$/GO/FTO before (a) and after (b) OER stability tested by chronoamperometry at 1.62 V vs RHE for 10 h.

Table S4. Comparison of the OER electrocatalytic performance of this work with FeCoNi LTH-related catalysts reported in the literature.

Electrocatalysts	η_{10} (mV)@ j_a=10 mA cm^{-2}	Tafel slop (mV dec^{-1})	Substrate	Ref.
FeCoNi LTH/$MnMoO_4$/GO	238	53	FTO	This work
NiCoFe LTH	184	37.8	[a]L-GCE	[1]
[b] FeCoNiBOx/PPy/rGO	290	47	GCE	[2]
NiCoFe LDHs	231	59	nickel foam	[3]
N–CoNiFe	318	72.2	GCE	[4]
CoNiFe LDH	196	49	stainless steel	[5]
FeCoNi-PBA	236	43.8	NF	[6]
NiCoFe LTHs	239	32	carbon fiber cloth	[7]
FeCoNi-P	239.5	55.87	Nf	[8]
FeCoNi-MoO_4	204	50.6	Nf	[9]
B_{10}–FeCoNi-LDH	169	93.72	NF	[10]
FeCoNi-S@NF	220	102.9	NF	[11]
FeCoNi LDH	240	58.37	NF	[12]
FeCoNi TMINs[c]	244	42.5	Titanium electrode	[13]
Act-CrFeCoNiCu	388	35.1	carbon cloth	[14]
$MnMoO_4$	239	50	NF	[15]
$NiCoO_2$/NiCo@C	329	61.8	-	[16]

a : L-shaped supporting glassy carbon electrode (L-GCE)
b: FeCoNi boride oxides immobilized on polypyrrole/reduced graphene oxide
c: Transition metal interstitial nitrides

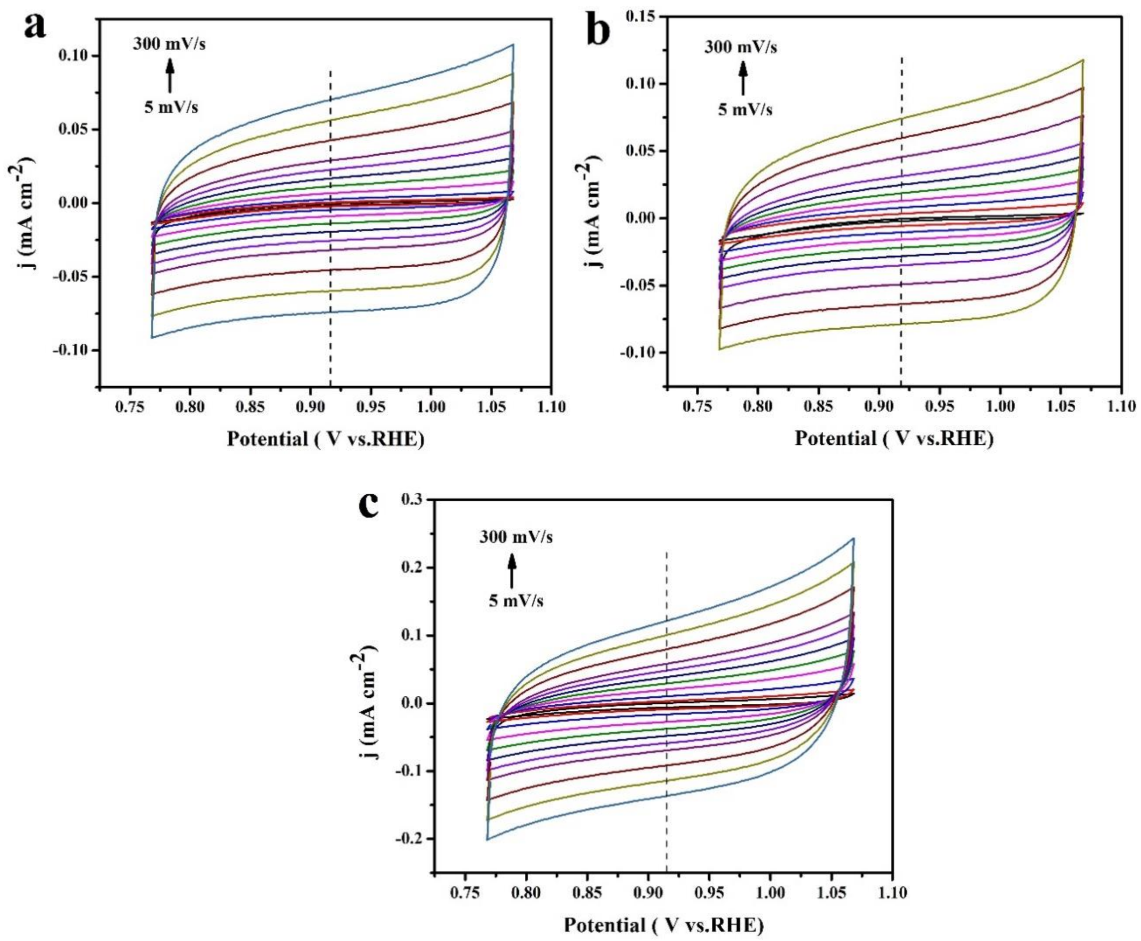

Fig. S9. CV curves of (a) FeCoNi LTH/FTO, (b) FeCoNi LTH/$MnMoO_4$/FTO, and (c) FeCoNi LTH/$MnMoO_4$/GO/FTO measured in 1.0 M KOH in the non-Faradaic potential window for HER.

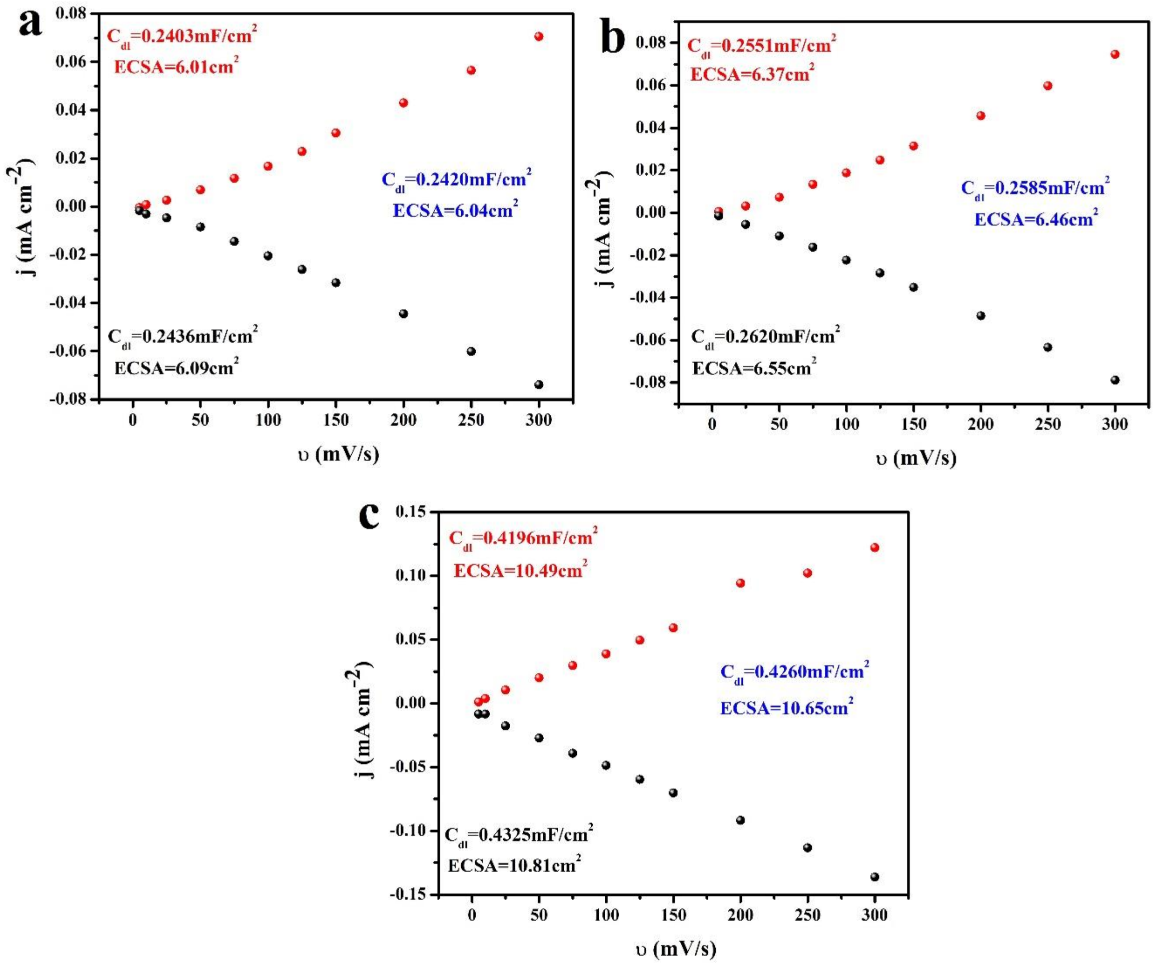

Fig. S10. Corresponding plots of the current density versus the scan rate for (a) FeCoNi LTH/FTO, (b) FeCoNi LTH/$MnMoO_4$/FTO, and (c)FeCoNi LTH/$MnMoO_4$/GO/FTO for HER.

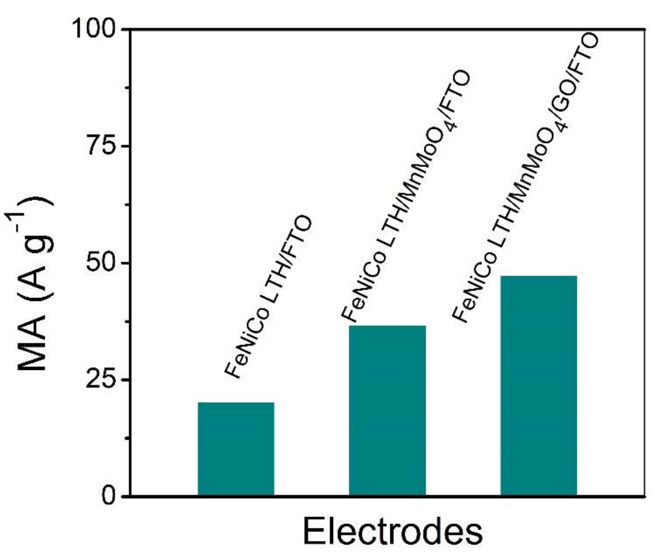

Fig. S11. Mass activity values at η = 300 mV for OER (a) and at η = 200 mV for HER (b).

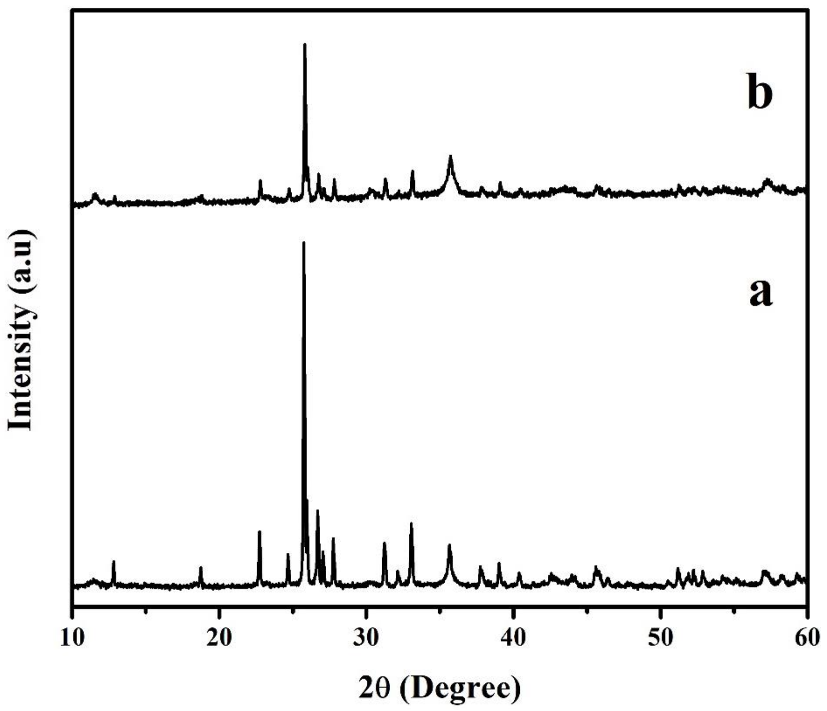

Fig. S12. XRD pattern for FeCoNi LTH/$MnMoO_4$/GO/FTO before (a) and after (b) 18 h stability in HER.

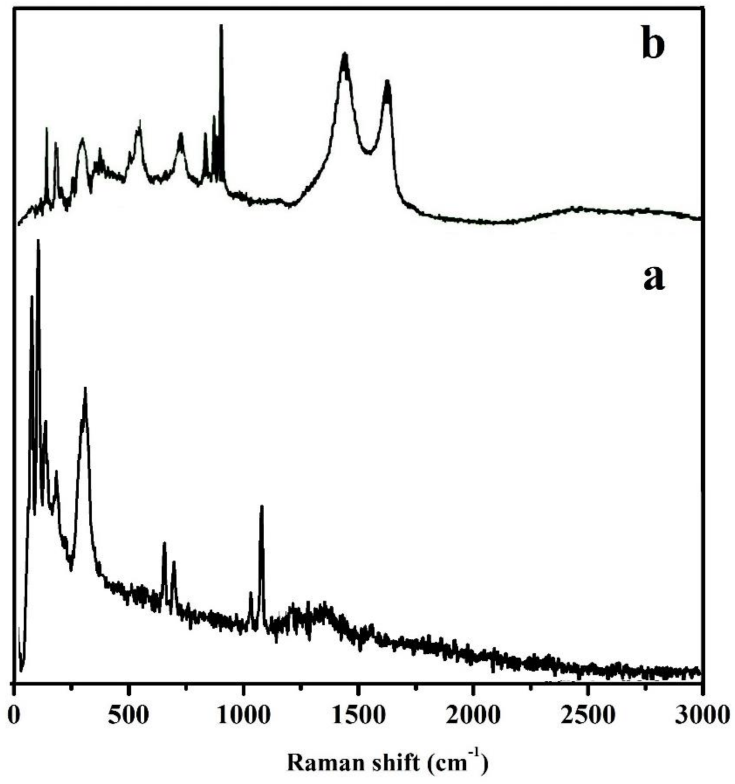

Fig. S13. Raman spectroscopy of FeCoNi LTH/$MnMoO_4$/GO/FTO after (a) and before (b) 18 h stability in HER.

Table S5. Comparison of the HER electrocatalytic performance of this work with FeCoNi LTH-related catalysts reported in the literature

Electrocatalysts	η_{10} (mV)@ j_a=10 mA cm^{-2}	Tafel slop (mV dec^{-1})	Substrate	Ref.
FeCoNi LTH/$MnMoO_4$/GO	92	46	FTO	This work
B_{10}–FeCoNi-LDH	56	39.26	NF	[10]
FeCoNi-S@NF	234	53.7	NF	[11]
NiCoFe LTHs/CFC	200	70	carbon fiber cloth	[7]
FeCoNi-S@NF	296	53.7	NF	[11]
MoS_2/$MnMoO_4$@Ti	153	80	Ti foil	[17]
$MnMoO_4$@CoS_2/MoS2/CC	71	78.1	CC	[18]
3DNi/Mn-MoO_4@3DNF	249	160	NF	[19]
$CoMoO_4$/$MnMoO_4$	153	86.28	NF	[18]
$NiCoO_2$/NiCo@C	61.9	42.6	-	[16]

Table S6. Detailed impedance fitting parameters (R_s, R_{ct}, CPE, n, and obtained from the equivalent circuit.

	Electrodes	R_s (Ω)	R_{ct} (Ω)	CPE ($\times10^{-3}$ Ω^{-1} s^n)	n	χ^2
OER	FTO	17.3	253.6	0.516	3.56	0.401
	GO/FTO	19.8	155.7	4.69	0.824	0.433
	$MnMO_4$/FTO	10.09	142.8	10.4	0.673	0.698
	FeCoNi LTH/FTO	4.42	76.6	13.43	0.52	0.551
	FeCoNi LTH/$MnMO_4$/FTO	19.2	57.1	16.5	0.814	0.852
	FeCoNi LTH/$MnMO_4$/GO/FTO	2.18	44.8	12.4	0.920	.0112
HER	FTO	39.8	208.7	7.31	1.1	0.686
	GO/FTO	22.5	136.8	8.9	0.793	0.532
	$MnMO_4$/FTO	8.65	93.9	7.91	0.501	0.625
	FeCoNi LTH/FTO	3.65	58.2	4.32	0.496	0.532
	FeCoNi LTH/$MnMO_4$/FTO	12.5	40.4	12.7	0.856	0.548
	FeCoNi LTH/$MnMO_4$/GO/FTO	11.9	15.30	25	0.924	0.209

Table S7. Comparisons of FeCoNi LTH/$MnMoO_4$/GO/FTO as bifunctional electrocatalysts for overall water splitting at 10 mA cm^{-2} with others in alkaline media.

**Electrocatalysts**	**The potential of j=10 mA cm^{-2} (V, vs.RHE)**	**Substrate**	**Ref.**
FeCoNi LTH/$MnMoO_4$/GO/FTO	1.56	FTO	This work
NiCoFe LTHs/CFC	1.55	carbon fiber cloth	[7]
FeCoNi-S@NF	1.70	NF	[11]
B_{10}–FeCoNi-LDH	1.54	NF	[10]
FeCoNi-Ni3C	1.664	-	[20]
FeCoNi LDH/Co LDH	1.63	NF	[21]
PtFeCoNiCu	1.48	CW	[22]
FeCoNi alloy	1.62	-	[23]
FeCo alloy/FeCoNi-Pi	1.56	NF	[24]
$MnMoO_4$/$CoWO_4$/NF	-	NF	[25]
AlMnFe-$NiMoO_4$/INF	1.58	NF	[26]
$NiFeSe_4$/$NiSe_2$	1.572	NF	[27]
MoS_2/$NiFe_2O_4$	1.69	silver	[28]

Table S8. Elemental composition of catalysts before stability testing determined by ICP-OES.

Sample	Fe (mg g^{-1})	Co (mg g^{-1})	Ni (mg g^{-1})	Mn (mg g^{-1})	Mo (mg g^{-1})	Total metal content (mg g^{-1})
FeCoNi LTH/FTO	132.10	89.50	93.60	< LOD	< LOD	315.20
FeCoNi LTH/$MnMoO_4$/FTO	103.30	72.70	81.00	113.40	139.00	509.40
FeCoNi LTH/$MnMoO_4$/GO/FTO	89.90	65.00	70.06	95.20	121.00	441.06

Volume of solution = 15 mL

To determine the elemental composition of the FeCoNi LTH/FTO, FeCoNi LTH/$MnMoO_4$/FTO, and FeCoNi LTH/$MnMoO_4$/GO/FTO nanocomposites after the overall water splitting stability test, measurements utilizing ICP-OES were conducted. The samples that were electrodeposited on FTO substrates were meticulously extracted from the electrochemical cell after chronoamperometry at 1.57 V for 31 h, and treated as described in section 2.2 of the manuscript. To determine the elemental composition of the catalysts that were prepared, the FTO electrode (with a geometric area of 1 cm^2) containing the deposited nanohybrid was submerged in 5 mL of aqua regia (composed of 3:1 HCl:HNO_3, v/v) and subjected to heating at 80 °C for a duration of 2 hours within a sealed Teflon vessel to ensure the complete dissolution of the catalyst layer. The resultant solution was then diluted to 15 mL using ultrapure water, filtered through a 0.22 µm PTFE filter to eliminate any undissolved residues, and subsequently analyzed using ICP-OES.

Table S9. Elemental composition of catalysts before stability testing determined by ICP-OES.

Sample	Fe (g mol^{-1})	Co (g mol^{-1})	Ni (g mol^{-1})	Mn (g mol^{-1})	Mo (g mol^{-1})	Fe:Co:Ni Molar ratio	Mn:Mo Molar ratio
FeCoNi LTH/FTO	2.37	1.52	1.59	< LOD	< LOD	1:0.64:0.67	-
FeCoNi LTH/$MnMoO_4$/FTO	1.85	1.23	1.38	2.06	1.45	1:0.67:0.75	1:0.70
FeCoNi LTH/$MnMoO_4$/GO/FTO	1.61	1.10	1.19	1.73	1.26	1:0.69:0.74	1:0.73

Volume of solution = 15 mL

For ICP-OES analysis, the catalyst-loaded electrodes (geometric area = 1 cm^2) were immersed in 15 mL of alkaline electrolyte during stability testing of overall water splitting at 1.57 V for 31 h (corresponding to Fig. 9b).

The initial mass loadings were approximately 0.28, 0.46, and 0.63 mg cm^{-2} for FeCoNi LTH, FeCoNi LTH/$MnMoO_4$, and FeCoNi LTH/$MnMoO_4$/GO FTO electrodes, respectively, measured the weight difference of the FTO electrode before and after the digestion.

Table S10. ICP-MS results of the electrodes, measured in mg L^{-1}, were established for electrodeposited films by digesting them with HNO_3 to analyze Fe, Co, Ni, Mn and Mo.

**Sample**	**Fe (mg L^{-1})**	**Co (mg L^{-1})**	**Ni (mg L^{-1})**	**Mn (mg L^{-1})**	**Mo (mg L^{-1})**	**Total dissolved metals (mg L^{-1})**
FeCoNi LTH/FTO	0.69	0.96	0.73	< LOD	< LOD	2.38
FeCoNi LTH/$MnMoO_4$/FTO	0.33	0.37	0.40	0.58	0.21	1.87
FeCoNi LTH/$MnMoO_4$/GO/FTO	0.24	0.31	0.27	0.36	15.00	1.33

Table S11. Relative metal leaching percentages after long-term stability testing.

**Sample**	**Fe (%)**	**Co (%)**	**Ni (%)**	**Mn (%)**	**Mo (%)**	**Total metal leaching (%)**
FeCoNi LTH/FTO	28.0	57.0	41.0	-	-	39.0
FeCoNi LTH/$MnMoO_4$/FTO	10.5	16.2	15.5	16.7	5.0	12.0
FeCoNi LTH/$MnMoO_4$/GO/FTO	6.4	11.2	9.3	9.0	3.5	7.3

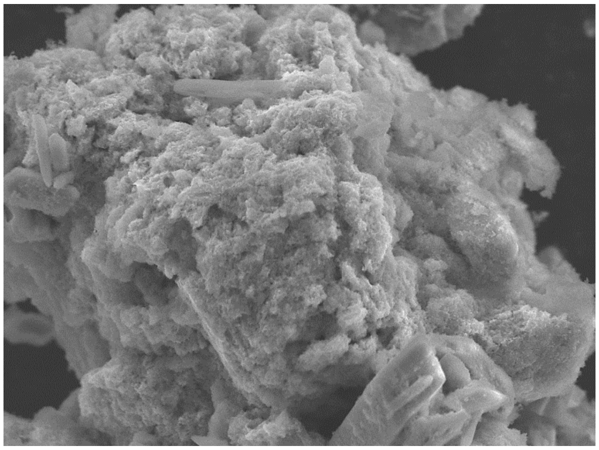

Fig. S14. FESEM image of FeCoNi LTH/$MnMoO_4$/GO/FTO after 31 h of overall water splitting stability at 1.57 V.

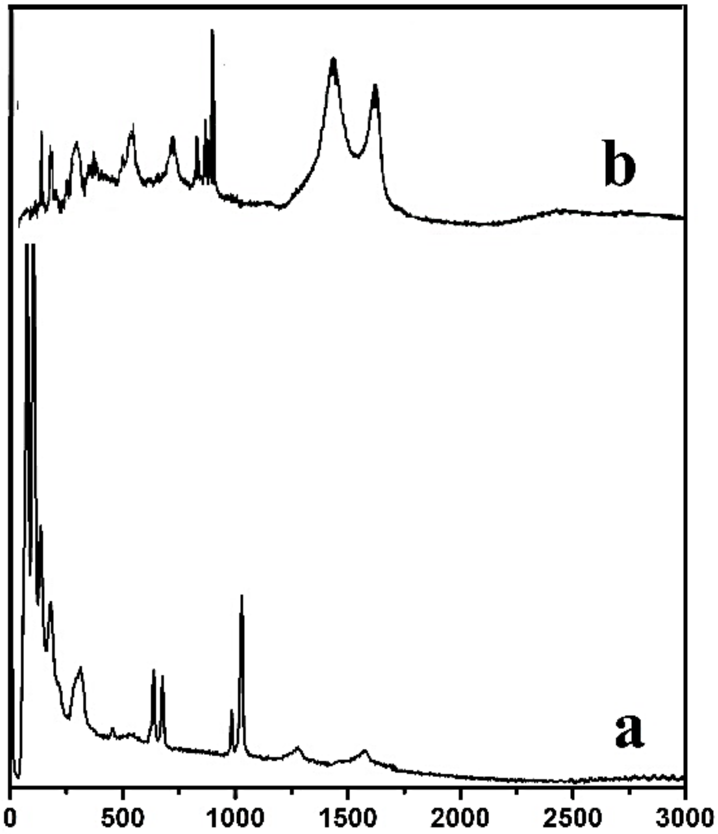

Fig. S15. Raman spectroscopy of FeCoNi LTH/$MnMoO_4$/GO/FTO after (a) and before (b) 31 h overall water splitting stability at 1.57 V.

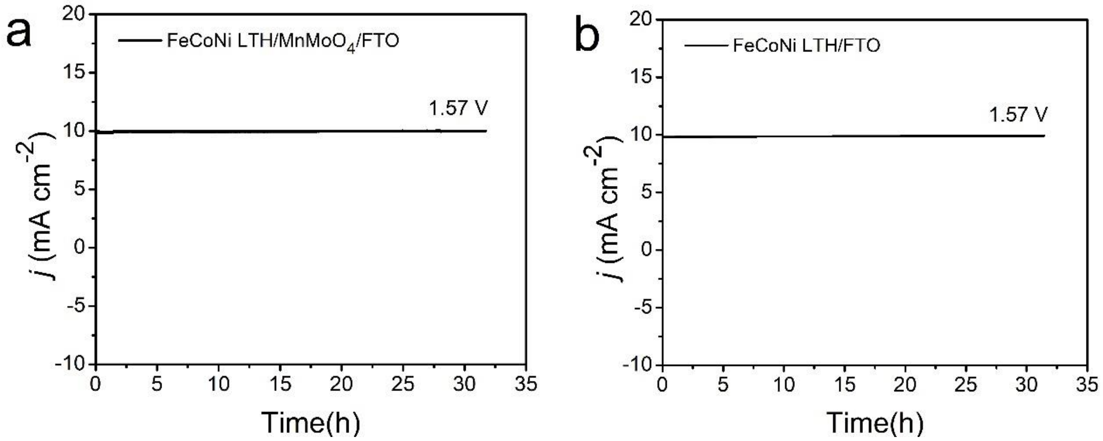

Fig. S16. Stability test i-t curve at 1.57 V for 31 h of: (a) FeCoNi LTH/$MnMoO_4$/FTO and (b) FeCoNi LTH/FTO.

References

[1] Z. Guo, Y. Wang, M. Li, S. Wang, F. Du, Vulcanization and acid etching of NiCoFe layered ternary hydroxides for enhancing oxygen evolution reaction, J. Alloys Compd. 832 (2020) 155012. https://doi.org/10.1016/j.jallcom.2020.155012.

[2] H. Mao, X. Guo, Y. Fu, H. Yang, Y. Zhang, R. Zhang, X.-M. Song, Enhanced electrolytic oxygen evolution by the synergistic effects of trimetallic FeCoNi boride oxides immobilized on polypyrrole/reduced graphene oxide, J. Mater. Chem. A. 8 (2020) 1821–1828. https://doi.org/10.1039/C9TA10756H.

[3] X. Wang, Y. He, Y. Zhou, R. Li, W. Lu, K. Wang, W. Liu, In situ growth of NiCoFe-layered double hydroxide through etching Ni foam matrix for highly enhanced oxygen evolution reaction, Int. J. Hydrogen Energy. 47 (2022) 23644–23652. https://doi.org/10.1016/j.ijhydene.2022.05.198.

[4] A.T.N. Nguyen, M. Kim, J.H. Shim, Controlled synthesis of trimetallic nitrogen-incorporated CoNiFe layered double hydroxide electrocatalysts for boosting the oxygen evolution reaction, RSC Adv. 12 (2022) 12891–12901. https://doi.org/10.1039/D2RA00919F.

[5] R.C. Rohit, A.D. Jagadale, S.K. Shinde, D.-Y. Kim, V.S. Kumbhar, M. Nakayama, Hierarchical nanosheets of ternary CoNiFe layered double hydroxide for supercapacitors and oxygen evolution reaction, J. Alloys Compd. 863 (2021) 158081. https://doi.org/10.1016/j.jallcom.2020.158081.

[6] T.X. Nguyen, K.-H. Yang, Y.-J. Huang, Y.-H. Su, O. Clemens, R.-K. Xie, Y.-J. Lin, J.-F. Lee, J.-M. Ting, Anodic oxidation-accelerated self-reconstruction of tri-metallic Prussian blue analogue toward robust oxygen evolution reaction performance, Chem. Eng. J. 474 (2023) 145831. https://doi.org/10.1016/j.cej.2023.145831.

[7] A.-L. Wang, H. Xu, G.-R. Li, NiCoFe Layered Triple Hydroxides with Porous Structures as High-Performance Electrocatalysts for Overall Water Splitting, ACS Energy Lett. 1

(2016) 445–453. https://doi.org/10.1021/acsenergylett.6b00219.

[8] Y. Guo, P. Wang, P. Li, M. Tang, H. Yin, D. Wang, A highly efficient and durable self-standing iron-cobalt-nickel trimetallic phosphide electrode for oxygen evolution reaction, J. Alloys Compd. 960 (2023) 170493. https://doi.org/10.1016/j.jallcom.2023.170493.

[9] W. Fan, C. Liu, H. Wang, J. Wu, S. Chen, W. Fang, C. Wu, Y. Quan, D. Wang, Y. Qi, FeCoNi molybdenum-based oxides for efficient electrocatalytic oxygen evolution reaction, J. Colloid Interface Sci. 662 (2024) 460–470. https://doi.org/10.1016/j.jcis.2024.02.104.

[10] L. Ma, X. Li, G. Zhang, Y. Zhang, H. Dong, J. Liao, R. Han, L. Zhang, Super-aerophobic B-doped coral-like hierarchical nanoarray electrode for overall water splitting, Int. J. Hydrogen Energy. 124 (2025) 251–262. https://doi.org/10.1016/j.ijhydene.2025.04.045.

[11] Z. Lin, L. Wang, T. Jia, X. Wang, C. Li, H. Wang, L. Li, Y. Zhou, C. Zhai, H. Tao, S. Li, ZIF-67-derived FeCoNi-LDH with a 3D nanoflower hierarchical structure for highly efficient oxidation of 5-Hydroxymethylfurfural and coupling seawater splitting hydrogen production, Chem. Eng. J. 481 (2024) 148429. https://doi.org/10.1016/j.cej.2023.148429.

[12] X. Yue, H. Liang, Y. Zhang, Y. Zhou, J. Wang, Z. Xie, P. Yang, Y. Ma, X. Li, Fabrication of FeCoNi LDH medium entropy hydroxide nanosheet for catalyzing oxygen evolution reaction, Appl. Catal. A Gen. 704 (2025) 120408. https://doi.org/10.1016/j.apcata.2025.120408.

[13] Y. Chen, J. Xu, L. Zhang, M. Jiang, Z.-H. Xie, P. Munroe, Interstitial nitrogen-driven surface reconstruction in FeCoNi coatings for enhanced OER activity and durability, Appl. Catal. B Environ. Energy. 382 (2026) 125955. https://doi.org/10.1016/j.apcatb.2025.125955.

[14] J. Ruiz Esquius, M. González-Ingelmo, M. López García, A.V. Laruelo, R. Santamaría, C. Blanco, V.G. Rocha, Self-supported FeCoNi(OHy)Ox oxy-hydroxide doped with Cr and Cu as robust low-loading catalyst for the alkaline oxygen evolution reaction, Int. J. Hydrogen Energy. 142 (2025) 596–605. https://doi.org/10.1016/j.ijhydene.2024.10.160.

[15] A. Rajput, A. Kumari, H.K. Basak, D. Ghosh, B. Chakraborty, Tracking the active phase and reaction pathway of the OER mediated by an MnMoO 4 microrod electro(pre)-catalyst, J. Mater. Chem. A. 12 (2024) 30810–30820. https://doi.org/10.1039/D4TA05985A.

[16] W. Li, Y. Chen, S. Liu, J. Tang, Enhanced electrocatalytic performance of carbon-coated NiCoO2/NiCo composites for efficient water splitting, Sci. Rep. 15 (2025) 12294. https://doi.org/10.1038/s41598-025-96880-0.

[17] J.G. Badiger, M. Arunachalam, R.S. Kanase, S.A. Sayed, K.-S. Ahn, J.-S. Ha, S.H. Kang, Highly stable MoS2/MnMoO4@Ti nanocomposite electrocatalysts for hydrogen evolution reaction, Int. J. Hydrogen Energy. 51 (2024) 156–168. https://doi.org/10.1016/j.ijhydene.2023.08.091.

[18] Q. Zhang, Z. Zhao, Self-protecting MnMoO4@CoS2/MoS2/CC composite heterojunction catalysts for stabilizing hydrogen evolution in alkaline solutions, J. Alloys Compd. 1040

(2025) 183445. https://doi.org/10.1016/j.jallcom.2025.183445.

[19] S.A. Ansari, Fabrication and evaluation of binder-free metal-molybdate electrodes for improved energy storage and hydrogen evolution applications, J. Power Sources. 646 (2025) 237185. https://doi.org/10.1016/j.jpowsour.2025.237185.

[20] J. Zhong, Y. Zhang, X. Huo, X. Zuo, H. Huang, X. Xu, N. Zhang, FeCoNi-Based Alloy Coatings as Low Overpotential Electrocatalysts for Alkaline Water Electrolysis, Chem. – An Asian J. 20 (2025). https://doi.org/10.1002/asia.202401086.

[21] H. Tian, Y. Kang, J. Zhang, Y. Wu, J. Zhang, S. Niu, Multiscale hierarchical ternary FeCoNi LDH/Co LDH architecture for efficient and durable water splitting, Int. J. Hydrogen Energy. 148 (2025) 149926. https://doi.org/10.1016/j.ijhydene.2025.06.116.

[22] F. Wang, S. Chen, Z. Tong, X. Liu, H. Zhou, Y. He, G. Shu, Y. Jin, W. Zheng, Electronegativity-Modulated PtFeCoNiCu High-Entropy Alloy Catalysts for Efficient HER and OER, ACS Appl. Mater. Interfaces. 17 (2025) 66617–66629. https://doi.org/10.1021/acsami.5c17409.

[23] Y. Chen, L. Yang, C. Li, Y. Wu, X. Lv, H. Wang, J. Qu, In situ Hydrothermal Oxidation of Ternary <scp>FeCoNi</scp> Alloy Electrode for Overall Water Splitting, ENERGY Environ. Mater. 7 (2024). https://doi.org/10.1002/eem2.12590.

[24] H. Li, Y. Xiao, X. Tian, X. Xing, M. Cao, Y. Wang, A crystalline/amorphous FeCo alloy/FeCoNi-Pi bifunctional electrocatalyst for efficient overall water splitting, Inorg. Chem. Front. 11 (2024) 3585–3595. https://doi.org/10.1039/D4QI00574K.

[25] C.-Y. Hsu, W.H. Hassan, E. Khudoynazarov, S.W. Ghori, D. Jumanazarov, S. Sundharam, S. Kumar, A.M.A. Mohamed, M.A. Diab, H.A. El-Sabban, I. Mahariq, High-performance multimetal broccoli-like structure MnMoO4/CoWO4/NF nanocomposite as a bifunctional electrocatalyst for efficient water splitting, J. Electroanal. Chem. 997 (2025) 119503. https://doi.org/10.1016/j.jelechem.2025.119503.

[26] H. Li, X. Gao, T. Wang, Z. Shi, J. Bai, Z. Wang, X. Wang, NiMoO4 nanosheets through co-doping of AlMnFe engineering for enhanced bifunctional electrochemical catalysis for overall water splitting, Fuel. 406 (2026) 137223. https://doi.org/10.1016/j.fuel.2025.137223.

[27] L. Mu, S. Qiu, G. Zhao, B. Zhang, W. Liao, N. Zhao, X. Xu, A high-efficiency NiFeSe 4 /NiSe 2 bifunctional electrocatalyst with outstanding oxygen evolution reaction and overall water splitting performance, J. Mater. Chem. A. 12 (2024) 1714–1724. https://doi.org/10.1039/D3TA06701G.

[28] N.A. Khan, G. Rahman, S.Y. Chae, A. ul H.A. Shah, O.S. Joo, S.A. Mian, A. Hussain, Boosting electrocatalytic hydrogen generation from water splitting with heterostructured MoS2/NiFe2O4 composite in alkaline media, Int. J. Hydrogen Energy. 69 (2024) 261–271. https://doi.org/10.1016/j.ijhydene.2024.05.042.
